# Metal–Organic Frameworks for CO_2_ Capture: Improving Adsorption Performance Through Modification Methods

**DOI:** 10.3390/nano16080454

**Published:** 2026-04-10

**Authors:** Hongyu Pan, Li Xu, Tong Xu, Bin Zhu

**Affiliations:** 1 Laboratory of Plasma Catalysis, Dalian Maritime University, Dalian 116026, China; 4032332165@smail.lnu.edu.cn (H.P.); tongxu@dlmu.edu.cn (T.X.); 2 Novel Energy Materials & Catalysis Research Center, Shanwei Institute of Technology, Shanwei 516600, China

**Keywords:** metal–organic frameworks, carbon capture adsorbents, modification, aperture adjustment, metal-ion doping, functional group doping, doped metal ions

## Abstract

Industrial emissions of large amounts of CO_2_ have seriously affected human health, making it imperative to reduce atmospheric CO_2_ concentrations. However, carbon capture technologies such as chemical absorption and membrane separation are still limited by high regenerative energy costs, corrosion, and low efficiency in diluting flue gas. Within this technological landscape, physical adsorption separation technology, due to its advantages such as a wide operating temperature range, low equipment corrosivity, and low regeneration energy consumption, has gradually become a research hotspot in carbon capture technology. The core of physical adsorption lies in finding high-quality adsorbents. Metal–organic frameworks (MOFs), with their ultra-high specific surface area, tunable pore structure, and abundant functionalization sites, are considered highly promising next-generation CO_2_ adsorbent materials. This review summarizes strategies for modifying MOFs to improve CO_2_ adsorption performance, focusing on aperture adjustment, doped metal ions, functional group doping, and computational screening. Performance enhancements are mechanism-dependent rather than simply additive. Moderate aperture adjustment and defect engineering can improve gas selectivity and CO_2_ capture capacity, while excessively narrow pores sacrifice available pore volume and gas diffusion. Doped metal ions, particularly in MOF-74 and related materials, can enhance CO_2_ capture capacity while controlling framework integrity and dopant composition. Functional group Doping remains an effective method for capturing low-partial-pressure CO_2_. Computational screening is shifting from ranking based on single adsorption capacity to a comprehensive consideration that includes humidity tolerance, stability, and regenerability. Overall, under industrial conditions, modified MOFs should be evaluated by balancing affinity, selectivity, capacity, stability, and energy efficiency. This review provides guidance for the rational design of MOF-based carbon capture adsorbents.

## 1. Introduction

**Figure 1:**
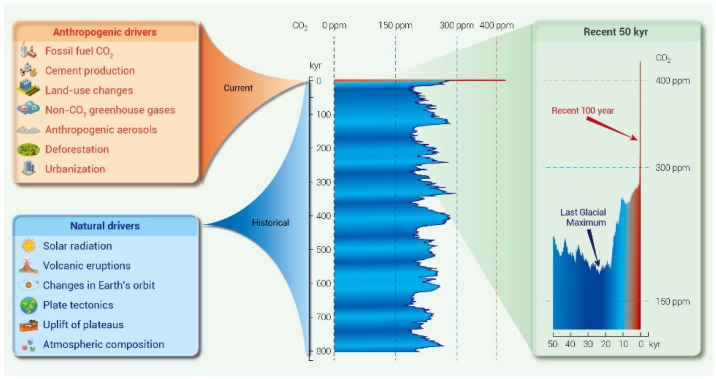
Evolution of atmospheric CO_2_ over the past 800,000 years (800 kyr), major climate change drivers, and greenhouse effect (reproduced with permission from reference [1]).

**Figure 2:**
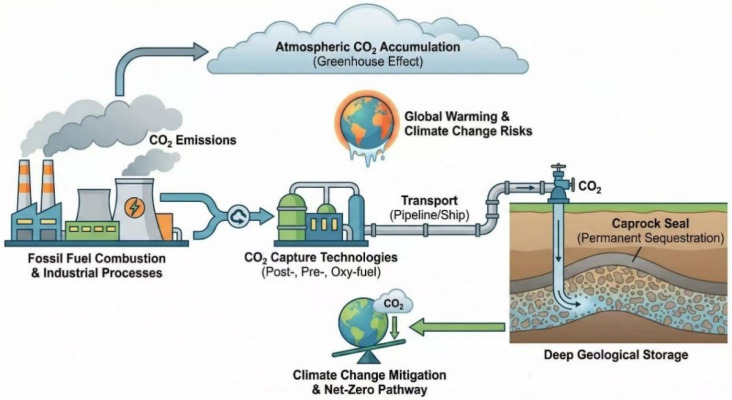
Schematic diagram of carbon capture and storage process.

Since the Industrial Revolution, the excessive reliance of human society on fossil fuels has led to a sharp increase in the atmospheric concentration of greenhouse gases, thereby triggering a climate crisis primarily manifested as global warming. Among the various greenhouse gases, CO_2_ is widely recognized as the dominant contributor to the greenhouse effect because of its large emission volume and long atmospheric lifetime. As of 2026, the atmospheric CO_2_ concentration remains above 420 ppm, as shown in Figure 1. This is accompanied by an increase in extreme weather events, including severe floods, prolonged droughts, and accelerated cryosphere retreat [1,2,3]. These changes have caused widespread environmental degradation, placed increasing stress on public health systems, and contributed to a rise in heat-related illnesses. Under the net-zero commitment established by the Paris Agreement, achieving deep reductions in CO_2_ emissions has become a central priority of global climate policy. As global warming intensifies and extreme weather events become more frequent, ecosystems are facing mounting pressure, while governments worldwide are under increasing urgency to accelerate energy and industrial transitions. The core challenge is to achieve rapid decarbonization while maintaining climate stability and economic development. In this context, carbon capture and storage (CCS) has emerged as a critical component of deep decarbonization strategies. Its importance is particularly evident in hard-to-abate sectors such as steel, cement, and chemicals, where full decarbonization remains exceptionally challenging due to unavoidable process emissions and technological constraints. Consequently, CCS is increasingly regarded as an indispensable pathway toward carbon neutrality [4], as shown in Figure 2. There are various methods for CCS, with chemical absorption of aqueous amines being the most mature option. However, its large-scale application is constrained by high regeneration energy demand, solvent degradation, and equipment corrosion [5]. Membrane separation and cryogenic processes are attractive in specific niche applications, but low CO_2_ partial pressures often compromise product purity and economic viability [6,7]. Calcium looping enables rapid high-temperature CO_2_ capture, yet the substantial energy consumption associated with repeated calcination remains a major unresolved challenge [8].

Physical adsorption separation technology has gradually become a research hotspot in the CCS field due to its advantages, such as a wide operating temperature range, low equipment corrosivity, low regeneration energy consumption, and no liquid waste discharge [9,10]. The core of adsorption separation technology lies in the performance of the adsorbent, but traditional adsorbents such as zeolites and activated carbon cannot maintain high performance and stability for CO_2_ adsorption under complex operating conditions. Among emerging porous materials, MOFs have attracted extensive attention because of their structural diversity and tunability. MOFs are crystalline porous coordination materials constructed from metal ions or clusters and organic linkers, forming highly ordered network structures. Their most notable advantages include exceptionally high specific surface areas and remarkable structural flexibility. Through rational framework design, both topology and local chemical environments can be tuned with near-atomic precision. Nevertheless, early MOFs often performed unsatisfactorily under realistic operating conditions. In humid flue gas, water vapor competes strongly with CO_2_ for adsorption sites, leading to significant losses in adsorption capacity and selectivity. Moreover, many MOFs exhibit insufficient hydrothermal stability and may undergo irreversible structural degradation during repeated adsorption–desorption cycles. These limitations have driven the field away from the mere discovery of entirely new frameworks toward the targeted modification of existing MOFs to enhance adsorption capacity, selectivity, and structural stability. However, a unified framework for classifying MOF modification strategies from an industrial applicability perspective remains incomplete.

In the paper, the most effective approaches for improving CO_2_ adsorption performance are categorized into three main strategies: aperture adjustment, doped metal ions, and functional group doping [11,12,13,14,15]. Doped metal ions mainly influence the local electrostatic field, the density of open metal sites, and the polarity of the framework [16]. Functional group doping controls the adsorption microenvironment through acid–base balance, dipole/quadrupole effects, hydrophilicity, and steric spectroscopy. In parallel, theoretical design has become equally important. Although computational screening does not alter the overall classification framework of modification strategies, it increasingly determines which modifications are worth experimental exploration. As a result, theoretical studies have evolved from merely interpreting adsorption mechanisms to guiding high-throughput screening and rational material design [14,15,17,18,19].

## 2. Synthesis of Metal–Organic Frameworks

### 2.1. Architectures and Design Objectives

For CO_2_ capture, MOF synthesis should be discussed in terms of the adsorptive function it enables rather than as a stand-alone preparative exercise [12,20]. Architectures based on one-dimensional metal-oxide chains, such as MOF-74 analogues, are valuable when a high density of open metal sites is desired because those sites can increase CO_2_ affinity at low pressure [16]. Zr-based clusters, exemplified by UiO-66, UiO-67, MOF-801, and MOF-808, are often selected when hydrothermal and chemical stability are central design requirements [21]. Cu-paddlewheel systems such as HKUST-1 can provide strong adsorption sites and high porosity, but their performance can be strongly influenced by guest activation and stability in humid environments [22,23]. Mesoporous MIL-101-type architectures offer large cages suitable for post-synthetic loading of amines or other functional molecules, but pore blocking becomes a major risk when the grafting density is high [24]. Ti-based MOFs and mixed-ligand systems occupy an intermediate position, where framework stability and chemical tunability are balanced against more complex synthesis and activation requirements [25,26]. MOF-74 derivatives, because of their accessible metal sites, are especially suitable for defect engineering and metal substitution [16,23,27,28,29,30,31,32,33,34,35,36]. Zr-based materials are more frequently used for post-synthetic functionalization because the underlying cluster remains stable during grafting or ligand exchange. Large-pore frameworks such as MOF-177 and MIL-101(Cr) are useful platforms for amine loading, but the same large-pore character that permits high loading can also dilute confinement effects that would otherwise improve selectivity [24]. For this reason, the most meaningful design question is rarely which MOF has the highest uptake, but instead which architecture best preserves useful pore volume while enabling a targeted change in low-pressure affinity, selectivity, or cyclic stability [21,37,38].

### 2.2. Synthetic Pathways and Morphological Control

**Figure 3:**
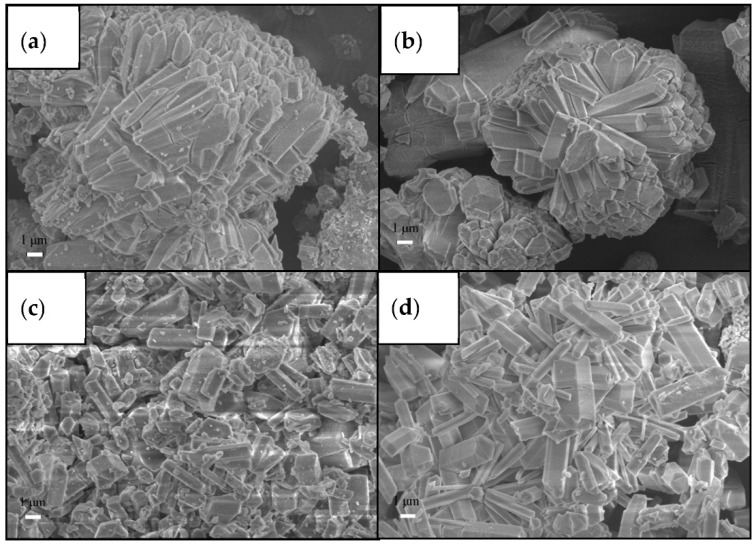
SEM images of Mg-MOF-74-N0 (**a**), Mg-MOF-74-N0.5 (**b**), Mg-MOF-74-N1 (**c**), and MgMOF-74-N2 (**d**) (reproduced with permission from reference [28]).

**Figure 4:**
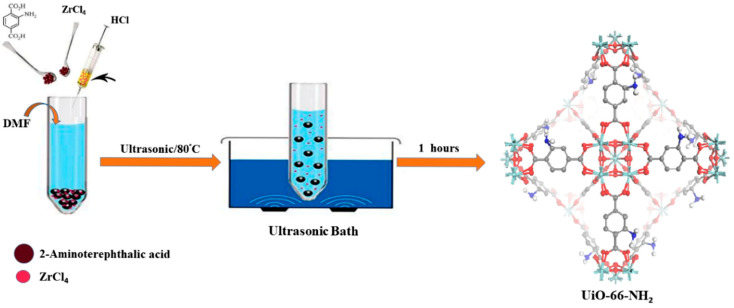
The preparation of UiO-66-NH_2_ by the sonochemical method (reproduced with permission from reference [39]).

Synthetic route and crystal growth control can influence CO_2_ capture even before any explicit post-modification is applied, because the route changes crystal size, defect concentration, accessible pore volume, and activation behavior. For Mg-MOF-74-Nx, H_4_DOBDC (0.674 g, 3.4 mmol) and Mg (NO_3_)_2_·6H_2_O (2.8 g, 10.9 mmol) were added to 300 mL of a DMF/EtOH/H_2_O mixed solution with a volume ratio of 15:1:1 and stirred until dissolved. Then, 0–2 equivalent amounts of NaAc were added to the mixture, and then the reaction liquid was placed in a crystallization kettle and reacted in an oven at 125 °C for 20 h. Finally, the morphology of Mg-MOF-74-Nx is shown in Figure 3 [27]. Shi et al. further showed that high-gravity synthesis could produce high-quality MOF-74-Co with a BET surface area of 1599 m^2^ g^−1^, and the small-sized MOF-74-Co sample with an average size of 78 nm exhibited a CO_2_ saturation capacity of 298 mg g^−1^ [31]. These results indicate that the synthesis step can already function as a modification method when it changes defect density or mass-transfer length scales in a controlled way. A similar conclusion arises in Zr-based systems. Kazemi et al. compared sonochemical and solvothermal UiO-66-NH_2_ and found that the sonochemically synthesized sample reached 3.2 mmol g^−1^ CO_2_ at 298 K and 1 bar, compared with 2.3 mmol g^−1^ for the solvothermal analogue, as shown in Figure 4. Under simulated flue-gas conditions, the same sonochemical sample displayed a CO_2_/N_2_ selectivity of 202 and a reported isosteric heat (Q_st_) above 80 kJ mol^−1^, while the capacity loss over eight cycles was only 0.6 mmol g^−1^ [39].

### 2.3. Economic Assessment and Feasibility

**Figure 5:**
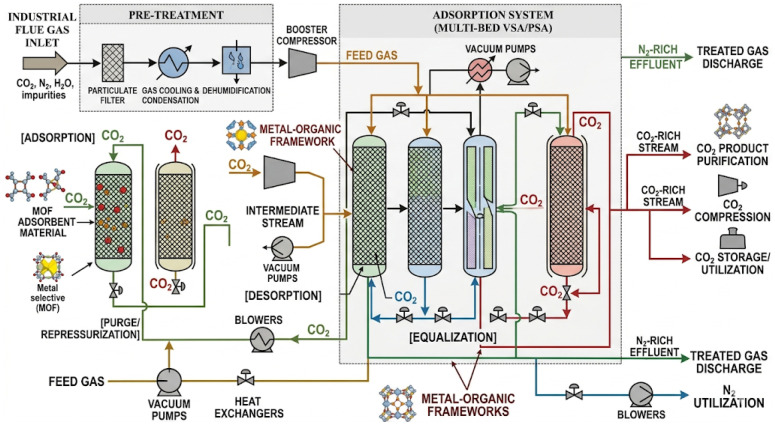
Schematic illustration of the industrial-scale MOF-based CO_2_ adsorption and separation process.

The industrial-scale process for CO_2_ adsorption and separation is illustrated in Figure 5. Flue gas first undergoes pretreatment and compression, then enters a multi-bed VSA/PSA unit packed with MOF adsorbents. Through cyclic adsorption, desorption, purge, and pressure equalization, CO_2_ is selectively captured, producing an N_2_-rich stream and a concentrated CO_2_ stream for further purification, compression, or storage. For industrial applications, however, adsorption performance alone is not enough; economic viability and practical feasibility must also be considered.

The economics of MOFs for physical CO_2_ adsorption should be evaluated from the viewpoint of industrial operation rather than equilibrium uptake alone. In practice, the value of an adsorbent depends on the total cost of synthesis, shaping, deployment, regeneration, and replacement during continuous use. A full cost assessment showed that MOF manufacturing still costs about 55 dollars per kilogram at 100 tons per year and 29.5 dollars per kilogram at 1 kiloton per year, whereas costs below 10 dollars per kilogram remain a long-term target that depends on cheaper ligands and more mature large-scale processing. At the process level, the minimum capture cost of MOF-based adsorption for wet flue gas was reported to be 91 versus 104.1 dollars per tonne of CO_2_ captured at 25 °C and 113.3 versus 146.9 dollars per tonne of CO_2_ captured at 40 °C under different process configurations [40]. These results show that industrial feasibility depends on the total cost per tonne of CO_2_ captured rather than on a single adsorption property. Within this framework, the value of aperture adjustment depends on whether pore-size control can be introduced without costly multistep post-synthetic treatment. However, these improvements are meaningful only if the added cost of fluorinated linkers, stricter synthesis control, or reduced pore volume is offset by lower adsorbent inventory or lower energy consumption. Similar considerations apply to structured adsorbents, since industrial systems require mechanically stable forms with high active-material utilization [41]. Metal-ion doping is often more attractive when the dopant can be introduced through a one-pot route while preserving the parent framework. Although such strategies can improve CO_2_ uptake and selectivity, their industrial value depends on whether the performance gain can offset higher precursor cost and tighter control in large-scale production. Functional-group doping often gives greater low-pressure improvement, but it is usually less economical because grafting or impregnation adds extra reagents and processing steps. Therefore, the most feasible strategy for industrial deployment is not necessarily the one with the highest affinity, but the one that best balances material cost, synthesis simplicity, shaping compatibility, and stable cyclic productivity under realistic conditions.

### 2.4. Regeneration and Desorption Considerations

For practical CO_2_ capture, the evaluation of MOFs should extend beyond adsorption capacity and selectivity to include the efficiency of CO_2_ release during regeneration. In cyclic operation, adsorption affinity, desorption kinetics, and regeneration energy are intrinsically linked, rather than independently optimized. When the interaction between CO_2_ and the framework is too weak, the working capacity under dilute flue-gas conditions becomes inadequate; when it is too strong, the energy demand for regeneration increases and CO_2_ desorption becomes less efficient. Accordingly, the objective of material design is not merely to maximize uptake, but to construct a pore environment that balances selective CO_2_ capture with rapid and reversible release.

This balance is governed to a large extent by the local structure of the adsorption environment. Moderate confinement, continuous diffusion pathways, and a controlled distribution of polar functionalities or open metal sites can sustain strong CO_2_ affinity without imposing an excessive desorption penalty. By contrast, overly restricted pore channels, excessive functional loading, or highly heterogeneous binding environments may enhance low-pressure uptake while simultaneously hindering mass transfer and increasing regeneration resistance. This trade-off is illustrated by diamine- and tetraamine-appended frameworks. In 2-ampd-Mg_2_(dobpdc), water-assisted cooperative adsorption enabled a high CO_2_ cycling capacity of 2.4 mmol g^−1^ with only a 100 °C temperature swing, demonstrating that a favorable adsorption mechanism can also support efficient regeneration [42]. Similarly, tetraamine-appended MOFs were shown to capture CO_2_ under humid conditions and to be regenerated directly with steam, indicating that strong adsorption affinity can remain compatible with practical desorption when the regeneration route is properly matched to the adsorption chemistry. CALF-20 offers a further example: as a durable physisorbent, it combines selective CO_2_ uptake with a low regeneration enthalpy and excellent steam stability over more than 450,000 cycles, highlighting the importance of framework robustness and the avoidance of water-dominated binding environments in maintaining regeneration efficiency.

A more regeneration-oriented perspective was provided by Alivand et al. [43], whose discussion is particularly relevant because it shifts attention from equilibrium capture alone to the structural factors governing CO_2_ release. They reported a water-dispersible Fe_3_O_4_@UiO-66-SO_4_ nanocatalyst for catalytic solvent regeneration, in which mesoporosity promoted molecular transport and sulfate-derived superacid sites accelerated desorption, reducing the overall energy consumption of CO_2_ capture by 44.7% with only 0.1 wt.% nanocatalyst while maintaining good recyclability. Although this system belongs to an absorption–regeneration process rather than a conventional fixed-bed physisorption cycle, it underscores a principle that is equally important for adsorption-based MOF design: the practical value of a material depends not only on how strongly it captures CO_2_, but also on how effectively its framework environment supports diffusion, desorption, low regeneration energy demand, and long-term cyclic efficiency.

## 3. Modification Methods

Modification is most useful when it is interpreted as a way to redistribute adsorption driving forces rather than as a generic means of improving all performance metrics simultaneously. In MOFs for CO_2_ capture, capacity, selectivity, Q_st_, diffusion rate, and regeneration penalty rarely increase together. A given modification may increase CO_2_ affinity while sacrificing available pore volume or may raise CO_2_/N_2_ selectivity by suppressing N_2_ uptake rather than by increasing CO_2_ loading. The subsections below therefore focus on how each modification method changes the balance between capacity and selectivity [13,15,21,37,44,45,46].

### 3.1. Aperture Adjustment

**Figure 6:**
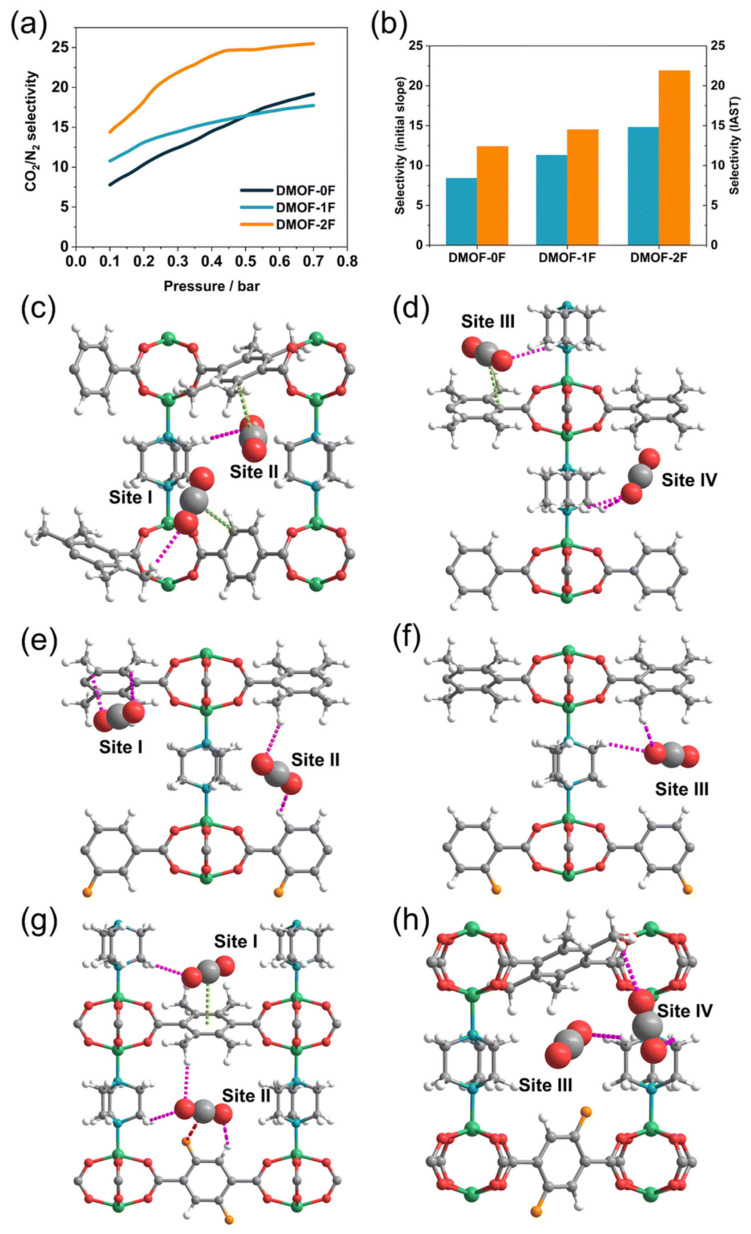
(**a**) Simulated IAST selectivity of CO_2_/N_2_ for DMOF-0F (dark green line), DMOF-1F (pale blue line), and DMOF-2F (orange line) under atmospheric CO_2_ concentration (i.e., 500 ppm of CO_2_ to N_2_). (**b**) CO_2_/N_2_ selectivity of three MOFs based on initial slope method (pale blue) and IAST method (orange). Simulated interactions between the framework and adsorbed CO_2_ molecules in (**c**,**d**) DMOF-0F, (**e**,**f**) DMOF-1F, and (**g**,**h**) DMOF-2F, where CO_2_ molecules with no effective interactions are emitted. CH/O(CO_2_), CF/C(CO_2_), and p/C(CO_2_) interactions are shown in pink, red, and light green dotted lines, respectively. (Reproduced with permission from reference [45]).

**Figure 7:**
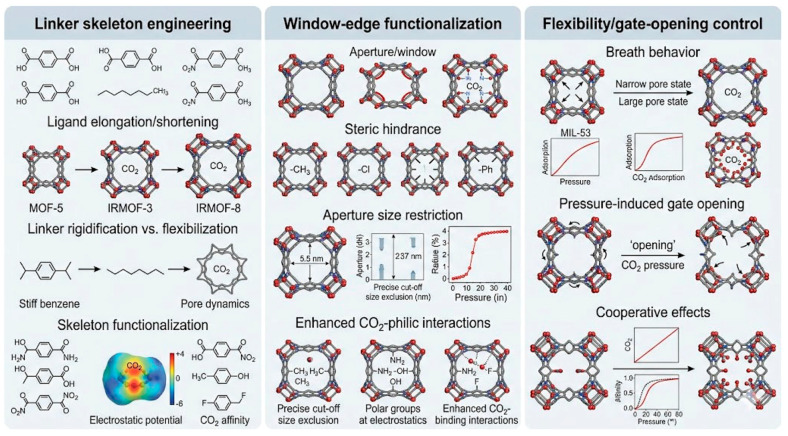
Three common methods in Aperture Adjustment.

Aperture adjustment is used in an operational sense to include linker substitution, defect engineering, and local pore-environment tuning that alter the effective window size or ultramicroporous confinement experienced by CO_2_. Di et al. reported a stepwise fluorination strategy in an isoreticular ultramicroporous MOF series to gradually reduce the pore size of DMOFs, where the CO_2_ uptake at 273 K and 1 bar increased from 4.55 mmol g^−1^ for DMOF-0F to 4.73 mmol g^−1^ for DMOF-1F and 4.79 mmol g^−1^ for DMOF-2F. The more informative changes, however, were in affinity and selectivity. Q_st_ increased from 19.3 to 20.2 and then 23.3 kJ mol^−1^, while the CO_2_/N_2_ selectivity rose from 8.4 to 11.3 and 14.8. At 0.3 bar, CO_2_/N_2_ selectivity increased from 12.4 to 14.5 and 21.9 across the same series as shown in Figure 6 [45]. This provides strong evidence that mild aperture narrowing combined with local polarity enhancement can improve low-pressure discrimination without a catastrophic loss of capacity. Defect engineering yields a related but distinct mechanism. By employing a chloride-assisted defect-engineering strategy, An et al. synthesized defect-rich hierarchical porous Mg-MOF-74, in which the weak coordination of Cl^−^ interfered with the regular assembly of Mg^2+^ and organic linkers, thereby generating ligand defects and hierarchical mesopores. As a result, the Qst increased from 36 to 46 kJ mol^−1^, the saturated CO_2_ uptake under ambient pressure rose by approximately 15%, and the CO_2_/N_2_ selectivity was enhanced by nearly 20-fold, which was attributed to the combined effects of stronger adsorption sites and accelerated mass transfer through hierarchical pore channels [29]. This is an important point because aperture adjustment is often assumed to mean only pore contraction. In practice, controlled defect creation can simultaneously create stronger local binding and shorter diffusion paths, provided that the framework remains intact and open metal sites remain accessible [47]. That mechanistic balance also determines whether aperture-engineered MOFs can leave the powder stage and enter real adsorption hardware. UTSA-16, whose small cages and narrow pore openings place CO_2_ in a size-matched confinement regime [48], has already been processed into pellets with 30 wt% loading in activated-carbon composites, giving 75% higher CO_2_ capacity and about 1000% higher CO_2_/N_2_ selectivity than the parent activated carbon pellet while retaining stability toward humid air, SO_x_, and NO_x_ [49], and has also been translated into 3D-printed monoliths that showed reproducible breakthrough behavior without detectable degradation even near 100% RH [50]. Related process-scale evidence has been obtained for shaped MIL-160(Al), where a three-bed six-step VPSA pilot delivered 90% CO_2_ purity and 92.7% recovery for a 15/85 vol% CO_2_/N_2_ feed, outperforming zeolite 13X under the same conditions [51]. Table 1 summarizes the adsorption performance of representative MOFs developed through aperture adjustment. The main limitation of aperture adjustment is that there is no monotonic relationship between narrower pores and better capture. When the window is narrowed too aggressively, diffusional resistance increases, and the gravimetric capacity can fall even if Q_st_ increases. The fluorinated MOF-801 illustrates the same point from another angle; partial modification can improve selectivity, whereas excessive functional loading can lower capacity because the window becomes too crowded. Aperture adjustment is therefore most convincing when it delivers a moderate shift in ultramicropore environment rather than an extreme reduction in accessible volume. Within aperture adjustment, linker skeleton engineering, window edge functionalization, and flexibility or gate opening control can be distinguished by how they regulate confinement rather than by whether they simply reduce pore size, as shown in Figure 7. Linker skeleton engineering changes the backbone length, shape, or rigidity of the linker and therefore shifts cage dimensions and window cross-section in a predictable way. Its main advantage is that pore contraction is encoded at the framework design stage, so stronger low-pressure CO_2_ discrimination can be achieved without relying on heavy post-synthetic loading, although excessive shortening can sacrifice pore volume and make the gain strongly topology dependent. Window edge functionalization acts more locally by decorating the aperture perimeter with polar or sterically active groups that adjust the electrostatic environment and the kinetic bottleneck seen by CO_2_ and competing gases. This route is particularly effective when small polar substituents strengthen quadrupole interactions while preserving diffusional access, but overdecoration can crowd the entrance and suppress useful uptake. Flexibility or gate opening control differs from both strategies because the effective aperture is not fixed but evolves during adsorption. By tuning linker dynamics, intraframework hydrogen bonding, or steric frustration, one can shift the balance between closed, intermediate, and open pore states so that CO_2_ triggers opening more readily than less interactive gases. In practice, the most useful aperture-adjusted MOFs are therefore those that combine moderate confinement, accessible transport pathways, and a controllable structural response under working pressures. For more examples of aperture adjustment, see Table 2.

### 3.2. Doped Metal Ions

**Figure 8:**
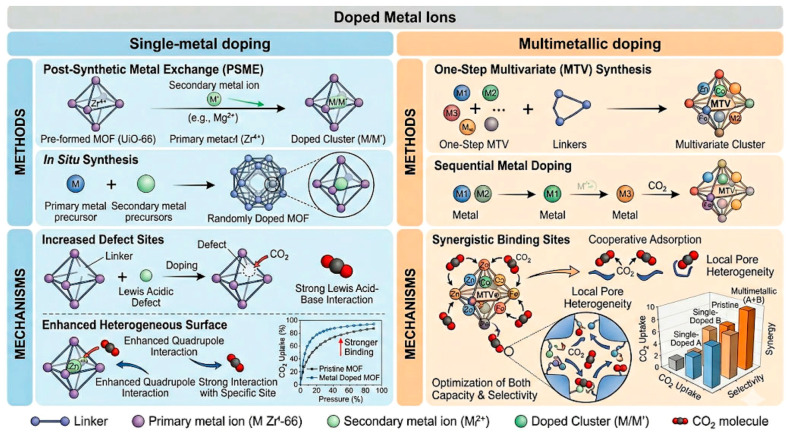
Two common methods in doped metal ions.

Doped metal ions alter the adsorption field more directly than aperture adjustment because the dopant can change charge density, polarizability, open-metal-site chemistry, and sometimes the framework topology itself. The usefulness of this route depends strongly on whether the parent framework survives the substitution and whether the dopant remains accessible after activation. Evidence in the recent literature suggests that the benefits of metal doping are real but composition-sensitive, and often restricted to a narrow doping window [23,25,34,35,36,69]. Metal-ion doping is divided into two categories: single-metal doping and multimetallic doping, as shown in Figure 8.

#### 3.2.1. Single-Metal Doping

**Figure 9:**
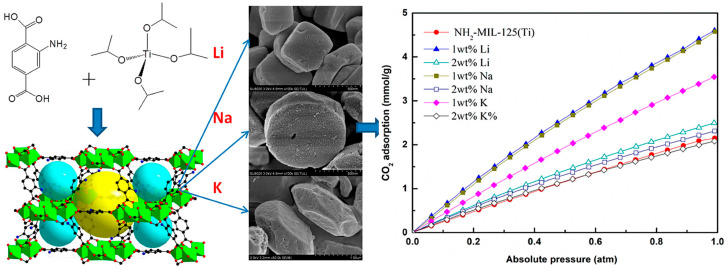
CO_2_ adsorption isotherms of NH_2_–MIL125(Ti) and xM@NH_2_–MIL125(Ti) at 293 K. (Reproduced with permission from reference [25]).

Only one dopant type or loading is varied on a common framework platform in single-metal-doped systems. In practice, this type of modification can be discussed in terms of two main material-centered categories, namely alkali-metal incorporation for electrostatic-field tuning and single-metal reinforcement of frameworks that already contain strong adsorption sites, while the dopant loading window and process-relevant applicability provide a final criterion for judging their practical value. Song et al. incorporated Li, Na, and K into NH_2_-MIL-125(Ti) and reported that 1 wt% Li- and Na-doped samples reached CO_2_ uptakes of 4.60 and 4.57 mmol g^−1^, respectively, at 293 K and 1 atm, whereas the 1 wt% K-doped sample reached 3.55 mmol g^−1^. The corresponding BET surface areas increased from 1038 m^2^ g^−1^ for pristine NH_2_-MIL-125(Ti) to 1470, 1451, and 1226 m^2^ g^−1^ for the Li-, Na-, and K-containing samples, as shown in Figure 9. The data suggest that alkali incorporation does not act only through simple surface-area increase; the identity of the cation shapes the adsorption field, and the heavier or more space-occupying dopant is not necessarily advantageous [25]. In the same general direction, Zhou et al. reported lithium-doped HKUST-1 with altered CO_2_ adsorption behavior, supporting the broader proposition that small alkali ions can tune the electrostatic environment of classical MOFs without complete structural replacement [23]. These studies are best understood as electrostatic-field-tuning cases, in which a single light metal species modulates local charge distribution and CO_2_ affinity without fundamentally reconstructing the parent framework. More pronounced gains have been observed in DOBDC-type frameworks. By contrast, the following studies belong to a second category, where single-metal doping acts mainly by reinforcing adsorption environments that are already strong because of open metal sites or highly accessible polar channels. Cui et al. modified Mg/DOBDC with alkali metals and reported that the best sample, 0.5 K-Mg/DOBDC, reached a dynamic CO_2_ adsorption capacity of 14.93 mmol g^−1^ at 0.1 MPa and 298 K, which was 3.44 times that of the parent Mg/DOBDC [32]. Ullah et al. similarly showed that alkali doping of MOF-74 increased BET surface area and pore size, and raised the dynamic CO_2_ adsorption capacity from 8.9 wt% to 11.68 wt% [33]. These studies suggest that single-metal doping can be especially effective when it reinforces strong adsorption sites already present in frameworks such as MOF-74 or Mg/DOBDC. At the same time, they illustrate the need to distinguish between equilibrium and dynamic data. Dynamic improvement is often more relevant to practical separations, but it can reflect changes in transport and site accessibility in addition to intrinsic thermodynamic affinity. For this reason, the final assessment of single-metal doping should not rely only on uptake enhancement, but also on whether the selected dopant level remains within a useful loading window and whether the modified framework can retain its advantage after shaping, humid-gas exposure, and cyclic operation. From an implementation perspective, however, published evidence for single-metal-doped MOFs still lies closer to engineering demonstration than to full pilot deployment. A Ni/DOBDC pellet from the same open-metal-site family used in metal-site tuning delivered a CO_2_ capacity of 3.74 mol kg^−1^ at 0.15 bar and a CO_2_/N_2_ selectivity of 38 in fixed-bed breakthrough tests, while still retaining significant performance at 3% RH [70]. Pelletized Mg-MOF-74 was likewise prepared without large capacity loss and dynamically benchmarked against zeolite 13X under low-pressure separation conditions [71]. Together with the CO_2_/H_2_O equilibrium and rate measurements reported for HKUST-1 and Ni/DOBDC [72], these results indicate that the practical value of a single dopant is realized only when the improved adsorption field survives pelletization, water competition, and cyclic packed-bed operation. The main caution is that higher dopant loading is not automatically better. Excessive loading can block pores, reduce crystallinity, or create chemically heterogeneous surfaces that complicate regeneration. Excessive single-metal doping can gradually convert a beneficial local perturbation into a structural and transport penalty. At moderate levels, isolated dopant species may strengthen the electrostatic field, improve the accessibility of adsorption sites, or increase the polarity of the pore environment. Once the loading exceeds the tolerance of the parent framework, however, the same dopant can begin to block pore apertures, occupy free volume, and disrupt the uniformity of the internal adsorption field. This often leads to slower CO_2_ diffusion, reduced site accessibility, and a loss of the balance between affinity and capacity. Overdoping may also lower crystallinity or create chemically heterogeneous regions, making adsorption sites less well defined and regeneration more difficult. In dynamic operation, these effects are even more pronounced because apparent performance then depends not only on equilibrium uptake, but also on mass transfer, water competition, and the ability of the framework to preserve its adsorption advantage after shaping and cycling.

#### 3.2.2. Multimetallic Doping

**Figure 10:**
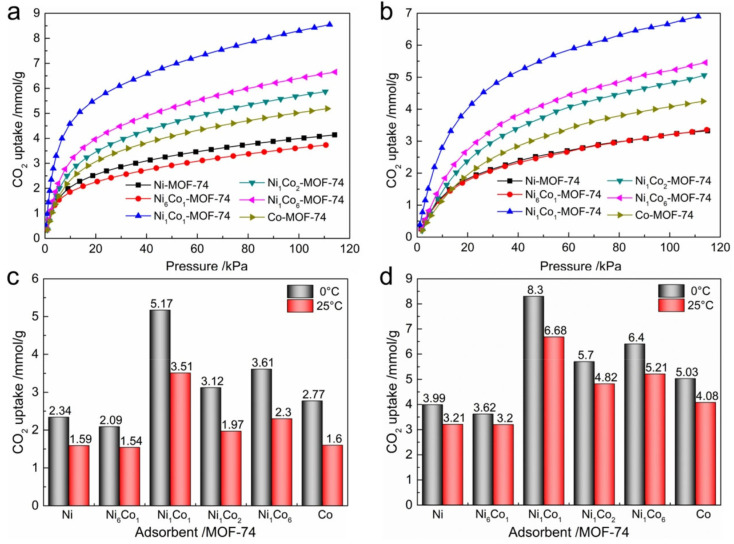
CO_2_ adsorption isotherms of Ni_x_Co_y_-MOF-74 at (**a**) 273 K and (**b**) 298 K; CO_2_ adsorption capacity of Ni_x_Co_y_-MOF-74 at (**c**) 0.15 bar and (**d**) 1.0 bar (reproduced with permission from reference [34]).

Multimetallic doping attempts to combine the benefits of two metal environments within one framework, usually to tune open-metal-site density, adsorption enthalpy, and local channel polarity. Multimetallic doping can be discussed through two closely related categories, namely composition-balanced bimetallic synergy in isostructural frameworks and low-level heterometal substitution that redistributes local adsorption fields while preserving the parent host, whereas the final criterion remains whether the mixed-metal composition stays within a useful substitution window and retains its advantage under process-relevant conditions. This approach is attractive in principle but only convincing when the framework topology is preserved, and the composition is well controlled. Chen et al. synthesized a series of bimetallic NiCo-MOF-74 materials by a microwave-assisted route and reported that Ni_1_Co_1_-MOF-74 reached a CO_2_ uptake of 8.30 mmol g^−1^ at 273 K and 1 bar. Under the same conditions, Ni-MOF-74 adsorbed 3.99 mmol g^−1^, Co-MOF-74 adsorbed 5.03 mmol g^−1^, Ni_6_Co_1_-MOF-74 adsorbed 3.62 mmol g^−1^, and Ni_1_Co_6_-MOF-74 adsorbed 6.40 mmol g^−1^, as shown in Figure 10 [34]. The CO_2_/N_2_ selectivity was reported to reach 34. The 1:1 composition outperforms both monometallic and off-stoichiometric bimetallic analogues, so the benefit was not simply more metal diversity but a narrow composition-dependent synergy. These results are representative of the first category, in which two metal environments cooperate most effectively only within a narrow stoichiometric range, indicating that multimetallic doping is valuable not because more metal species are present, but because a balanced combination can optimize the density, accessibility, and polarity of adsorption sites simultaneously.

Recent environmentally oriented syntheses of mixed-metal MOF-74 derivatives lead to the same conclusion. Kazemi et al. reported a 1:1 CoNiMOF-74 with CO_2_ uptakes of 7.55 mmol g^−1^ at 298 K and 9.36 mmol g^−1^ at 278 K, a Q_st_ of 40.7 kJ mol^−1^, CO_2_/N_2_ selectivity around 27.5, and stable performance over 10 cycles [35], together with the simulated-flue-gas selectivity and cycling stability already observed for CoNiMOF-74, these results suggest that the most realistic near-term route for multimetallic MOFs is shaped or packed adsorber validation under cyclic and compositionally relevant conditions. Zhang et al. prepared Nix/Mgx-MOF-74 and found that Ni0.11/Mg0.89-MOF-74 reached 7.02 mmol g^−1^ CO_2_ capacity and a CO_2_/N_2_ selectivity of 20.50, corresponding to improvements of 10.2% in capacity and 18.02% in selectivity relative to Mg-MOF-74 [36]. A second category is represented by systems in which a limited amount of the second metal is introduced to perturb the parent framework selectively rather than to create a fully equivalent dual-metal lattice, so that improvement arises from controlled redistribution of adsorption enthalpy, open-metal-site character, or channel polarity while structural retention remains the essential requirement. A broader modification strategy combines metal doping with chemical loading. Khan et al. investigated Mg-doped MOF-199 and showed that only up to 2 mol% Mg substitution preserved the framework. The 2 mol% Mg-doped material adsorbed 8.61 mmol g^−1^ CO_2_ at 273.15 K and 1 bar, representing a 17.78% increase over pristine MOF-199. When 40 wt% polyethyleneimine was subsequently introduced, CO_2_ adsorption selectivity increased by 37.90% [69]. Low-level Mg substitution improved uptake while preserving the host, and polymer loading then amplified selectivity. Accordingly, the practical value of multimetallic doping should be judged not only by the highest equilibrium uptake or selectivity, but also by whether the optimized metal ratio remains inside a stable composition window and whether the mixed-metal framework can preserve its adsorption advantage after shaping, repeated cycling, and dynamic separation testing. From an implementation viewpoint, however, the published evidence for multimetallic doping still lies mainly at the level of packed-bed and cyclic demonstrations rather than dedicated pilot plants. A bimetallic Mg-Ca/DOBDC adsorbent reached a dynamic CO_2_ uptake of 10.92 mmol g^−1^ at 0.1 MPa and 25 °C, while retaining 82.5% of its adsorption capacity after ten adsorption–desorption cycles, indicating that dual-metal-site tuning can survive repeated fixed-bed operation [73]. Dynamic separation evidence has also been reported for MIL-101(Cr-Al), where the CO_2_/CH_4_ dynamic selectivity increased from 1.82 to 4.2 at 15 bar with a space time of 1.12 min [74]. For more examples of multimetallic doping, see Table 3.

### 3.3. Functional Group Doping

**Figure 11:**
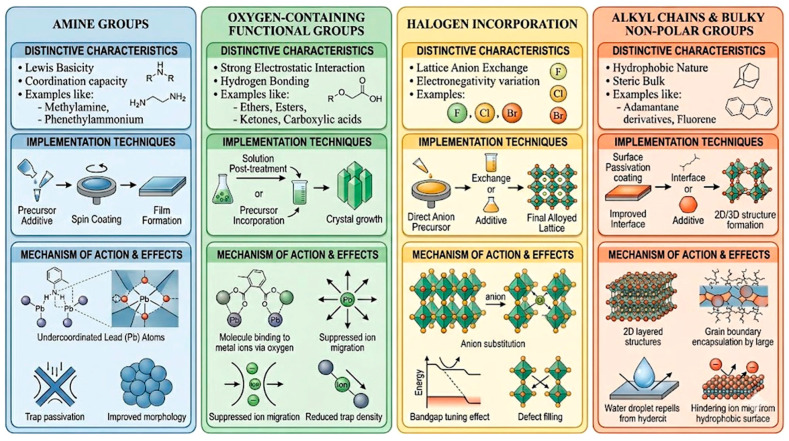
Four common methods in functional group doping.

Functional-group doping is one of the broadest and most heavily used strategies in MOF-based CO_2_ capture because it can be performed by linker design, post-synthetic grafting, covalent anchoring, or guest loading. This category refers to deliberate introduction of chemical moieties that reshape the adsorption microenvironment by changing local polarity, acid–base character, hydrogen-bonding capacity, steric hindrance, or hydrophobicity. The evidence base is uneven across functional-group classes. Amines are strongly supported by quantitative low-pressure data. Oxygen-containing groups usually produce more moderate gains unless they are part of a multistep architecture, as shown in Figure 11.

#### 3.3.1. Amine

**Figure 12:**
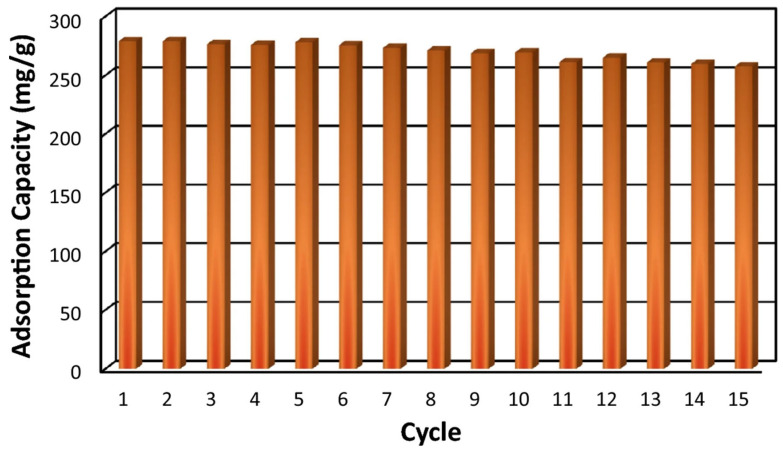
Regenerability of MH-20% sample for consecutive CO_2_ adsorption process (reproduced with permission from reference [89]).

Amine modification remains the most consistently validated route for improving low-pressure CO_2_ capture because it can introduce specific acid–base interactions and, in some cases, chemisorption-like behavior. The quantitative effect depends strongly on amine size, loading level, and pore geometry. Mutyala et al. incorporated tetraethylenepentamine into UiO-66 and found that 30TEPA/UiO-66 adsorbed 3.70 mmol g^−1^ CO_2_ at 348 K and 1 bar. The value is notable because it was achieved under relatively elevated temperatures, where physisorption-dominated MOFs often lose capacity [84]. Pirzadeh et al. compared aminated UiO-66 and Cu_3_(BTC)_2_ and reported CO_2_ uptakes of 3.32 and 3.86 mmol g^−1^, respectively, at 298 K and 1 bar. Under a 15/85 vol% CO_2_/N_2_ mixture, the CO_2_/N_2_ selectivities were 120 for NH_2_-UiO-66 and 53 for NH_2_-Cu_3_(BTC)_2_, while NH_2_-Cu_3_(BTC)_2_ showed a Q_st_ of 43 kJ mol^−1^ [85]. These results indicate that amination does not act uniformly across frameworks; the same functional group can give higher uptake on one platform and higher selectivity on another, depending on pore architecture and site accessibility. Large-pore frameworks make it possible to increase amine loading, but the gain is useful only if pore blocking remains limited. Gaikwad et al. functionalized MOF-177 with TEPA and showed that 20% TEPA-MOF-177 increased the CO_2_ capacity by 4.8 times relative to the parent at 298 K, while reaching 4.6 mmol g^−1^ at 328 K. The high-temperature performance is especially important because it suggests that strong amine-CO_2_ interactions compensated for the loss of weak physisorption that would otherwise occur at elevated temperature [86]. A similar principle was demonstrated by Jun et al. in MOF-808 functionalized with ethyleneamines. The MOF-808-TEPA sample adsorbed about 2.5 times as much CO_2_ as pristine MOF-808 at 15 kPa and delivered a CO_2_/N_2_ selectivity of 256, about seven times that of the parent [87]. The study provides strong evidence that polyamine loading is particularly effective under low-pressure conditions relevant to flue gas. Recent Zr-based studies reinforce the same point while also clarifying the limits of overfunctionalization. Nam et al. functionalized MOF-808 through an EDTA-assisted route followed by trisamine introduction and reported that MOF808-EDTA-TREN reached a CO_2_/N_2_ selectivity of 519 at 100 kPa, with stable performance over five runs. But a moderate degree of TREN functionalization is optimal, because increasing TREN loading raises the density of amino sites and strengthens low-pressure CO_2_ capture, excessive grafting markedly reduces BET surface area and pore volume from 2087 m^2^ g^−1^ and 0.70 cm^3^ g^−1^ for pristine MOF-808 to 118 m^2^ g^−1^ and 0.09 cm^3^ g^−1^ for MOF808-EDTA-TREN(0.5), which severely limits the CO_2_ adsorption environment of the skeleton [88]. On the higher-pressure side, Esfahani et al. modified Zr-BTC with NH_2_-containing mixed ligands and observed a maximum equilibrium CO_2_ capacity of 369.11 mg g^−1^ at 298 K and 9 bar for the sample containing 20 wt% NH_2_. The same modification increased specific surface area and pore volume by 15% and 6%, respectively, and after 15 cycles, the capacity decreased only from 279.05 to 257.56 mg g^−1^, as shown in Figure 12 [89].

Practical translation of amine-functionalized MOFs has also begun to move beyond powder tests, although fully dedicated pilot-scale reports remain limited. Monolith-supported mmen-Mg_2_(dobpdc) delivered CO_2_ uptakes of 2.37 mmol g^−1^ for 10% CO_2_ and 2.88 mmol g^−1^ for pure CO_2_, with excellent multicycle performance in a scalable low-pressure-drop honeycomb contactor [90]. A related 2-ampd-Mg_2_(dobpdc)/PES hollow-fiber module contained up to 70 wt% MOF and preserved 98% of the pure-MOF uptake while showing breakthrough behavior consistent with the parent framework and reduced pressure drop. At the process level, tetraamine-appended frameworks were further shown to capture CO_2_ efficiently under humid natural-gas-flue-gas conditions and could be regenerated directly with steam, while subsequent fixed-bed process analysis for an approximately 600 MW NGCC plant indicated that such materials can be embedded into temperature-swing adsorption systems with capture costs only about 30% higher than solvent capture under realistic heat-recovery assumptions [91].

Aminosilane-functionalized Ti-based MOFs and aminosilane-functionalized UiO-67 have also been reported to improve selective CO_2_ capture, but the mechanistic value of these studies lies less in reporting the highest absolute uptake and more in showing that tether length, grafting mode, and the ratio of amine to free pore volume are decisive. Across the recent literature, the strongest general conclusion is that amines are most effective for low-pressure capture when they are introduced at a loading that increases site-specific affinity without collapsing diffusional access. Very high Qst values can be advantageous for selectivity, but they may impose a larger regeneration penalty. Thus, the best-performing amine-modified MOFs are not necessarily those with the highest amine loading, but rather those in which amine accessibility and residual pore connectivity remain balanced.

#### 3.3.2. Oxygen-Containing Functional Groups

Compared with amines, oxygen-containing groups usually deliver more moderate but still useful changes in CO_2_ adsorption because they can increase local polarity, create hydrogen-bond acceptor sites, and alter pore wetting behavior without always causing the strong pore blocking associated with bulky polyamines. MFM-300(V^III^) contains bridging –OH groups lining the pore channels. The –OH groups strengthen host–guest interactions by providing a specific hydrogen-bonding site for CO_2_ and increasing the adsorption enthalpy, while also slightly narrowing the pore environment to favor stronger confinement. As a result, MFM-300(V^III^) shows a CO_2_ uptake of 6.0 mmol g^−1^ at 298 K and 1 bar, whereas MFM-300(V^IV^) adsorbs only 3.54 mmol g^−1^ under the same conditions, corresponding to a 41% increase. At 273 K and 1 bar, the uptake also rises from 6.56 to 8.6 mmol g^−1^, an improvement of about 31%. The Q_st_ of adsorption is higher for the hydroxyl-bearing material, confirming that pore hydroxylation enhances CO_2_ affinity and overall adsorption performance [92]. Park et al. modified MOF-808 stepwise and reported CO_2_/N_2_ selectivities of 40 for pristine MOF-808, 48 for MOF-808-EDTA, 19 for MOF-808-EDTA-ED, and 197 for the reduced derivative MOF-808-EDTA-ED-R, measured at 298 K and 1 bar. Clearly, the adsorption performance improved and then declined again because the functionalization of oxygen-rich EDTA provides numerous carboxyl-related sites, enhancing electron transfer between the framework and maintaining acid–base balance at the adsorption sites, thus providing an excellent adsorption environment for CO_2_ separation. However, not every additional functionalization is beneficial. The intermediate product MOF-808-EDTA-ED forms a high concentration of amide groups, leading to a decrease in the concentration of basic sites. Inappropriate chemicals can reduce releasable porosity or create unfavorable adsorption environments. The final product, MOF-808-EDTA-ED-R, transforms into an amine-functionalized material, significantly improving separation performance, as shown in Table 4 [93]. Oxygen-containing groups are therefore better understood as controlled polar modifiers than as inherently strong CO_2_-binding sites. At the implementation level, oxygen-containing pore environments appear attractive precisely because they usually preserve moderate binding strength and therefore avoid the excessive regeneration penalty often associated with stronger chemisorptive routes. Dedicated pilot-scale reports for this subclass are still scarce, but hydroxyl-lined frameworks have already moved into realistic packed-bed demonstrations. MFM-300(Fe), in which μ2-OH groups are preferred CO_2_ binding sites, was synthesized on a 1000-fold larger scale and achieved packed-bed CO_2_/N_2_ breakthrough separation with a selectivity of 21.6. Earlier, MIL-53(Al, PVA) pellets also demonstrated effective CO_2_/CH_4_ column separation, showing that hydroxyl-containing frameworks can be shaped without losing their practical adsorption function [94]. These results suggest that oxygen-containing functionalization is especially relevant when the target is not the highest possible affinity, but a robust adsorbent that can be pelletized, cycled, and integrated into fixed-bed contactors with acceptable mass transfer and regeneration behavior.

The broader evidence base reviewed by Lee et al. similarly suggests that non-amine polar groups can increase adsorption capacity, selectivity, or Q_st_, but the gain is often smaller than that of well-positioned amines and more sensitive to framework geometry. In many cases, oxygen-containing groups serve best as enabling elements for multistep modification, for example, by anchoring secondary functionalities or stabilizing a post-synthetic transformation [44]. That interpretation is consistent with the MOF-808 series above, where the oxygen-rich intermediate was beneficial but not transformative on its own. For this reason, oxygen-containing functionalization should be regarded as a moderate-strength strategy, especially valuable when a milder increase in affinity is preferred over the stronger regeneration penalty that can accompany polyamine loading.

#### 3.3.3. Halogen Incorporation

**Figure 13:**
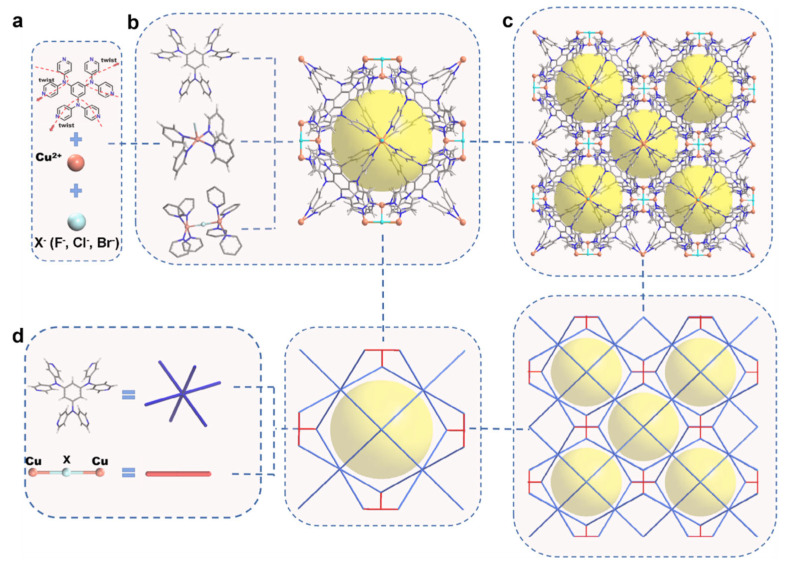
Assembly process of LNU-H1, LNU-H2, and LNU-H3. (**a**) Every two teeth in the hexadentate ligand are twisted around N and bind to Cu^2+^ cations and X^−^ (X = F, Cl, and Br) anions. (**b**) The coordination environment of mononuclear copper and fluorine atoms and the assembled 12-hedral cage. (**c**) An infinitely extended 3D network assembled from individual cages. (**d**) Simplified topological graph of LNU-H1, LNU-H2, and LNU-H3. (Reproduced with permission from reference [95]).

Halogen incorporation, especially fluorination, has become one of the most persuasive non-amine strategies for increasing CO_2_ selectivity in MOFs. Halogen-modified MOFs tend to exhibit stronger electrostatic or dipole-quadrupole interactions with CO_2_, altering electronic properties while enhancing hydrophobicity. Furthermore, the introduction of halogens can also modulate pore characteristics. In the isoreticular fluorinated DMOF series reported by Di et al., stepwise fluorination increased the CO_2_ uptake only modestly from 4.55 to 4.79 mmol g^−1^ at 273 K and 1 bar, but it increased Q_st_ from 19.3 to 23.3 kJ mol^−1^ and raised CO_2_/N_2_ selectivity at 0.3 bar from 12.4 to 21.9, as shown in Table 5 [45]. This pattern is important because it shows that fluorination may be more valuable for low-pressure discrimination than for maximizing total gravimetric capacity. Venturi et al. reached a similar conclusion in fluorinated MOF-801 analogues. By increasing the incorporation of fluorinated units, the authors raised Q_st_ up to 30 kJ mol^−1^ and obtained a CO_2_/N_2_ selectivity of 41 in the partially fluorinated analogue PF-MOF. However, complete fluorination reduced the adsorption capacity because the bulky fluorinated moiety narrowed the pore windows too strongly; at the same time, the interaction between CO_2_ and hydrogen bonds led to changes in the optimal adsorption site of CO_2_-MOF [46]. It demonstrates both the benefit and the limit of halogen incorporation within one material family. Fluorination is most effective when it enhances local electrostatics without imposing excessive steric crowding. In addition to fluorination, to address the bulky disadvantage of MOFs, Han et al. incorporated Cu-F/Cl/Br-Cu into LNU-H1/2/3, as shown in Figure 13. At the same time, Cu-F/Cl/Br-Cu replaced the original organic ligands, acting as a blocker to eliminate the one-dimensional channels with scarce polar sites, thus forming single-type-cage cage-base MOFs [95]. Halogen incorporation in MOFs often coexists with aperture adjustment, because halogen substituents not only modify the pore-surface polarity and electronic environment but also alter the effective window size and channel shape. This dual effect can synergistically enhance CO_2_ adsorption by strengthening host–guest interactions while simultaneously improving molecular sieving and diffusion selectivity.

#### 3.3.4. Alkyl Chains and Bulky Non-Polar Groups

**Figure 14:**
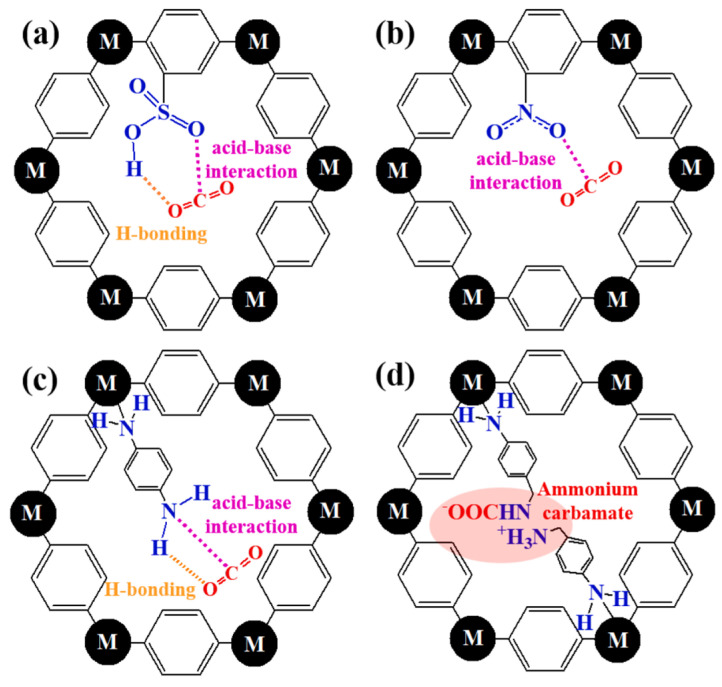
Plausible mechanisms of CO_2_ adsorption over (**a**) -SO_3_H, (**b**) -NO_2_, (**c**) aryl-NH_2_, and (**d**) alkyl-NH_2_ (reproduced with permission from reference [24]).

In principle, nonpolar groups can indirectly improve selectivity by altering pore constraint, restricting the window for N_2_ molecules to enter the pore, or controlling the configuration of adjacent polar groups, but they generally do not provide strong interactions with CO_2_ [44,96]. Lee et al. functionalized MIL-101(Cr), where alkyl-NH_2_, aryl-NH_2_, -SO_3_H, and -NO_2_ groups were compared under matched conditions. The reported performance order was alkyl-NH_2_ > -SO_3_H > aryl-NH_2_ > -NO_2_, indicating that local chemical environment and flexibility matter more than nominal functional-group identity alone. However, even the excellent performance of alkyl-NH_2_ failed to isolate a purely nonpolar effect, as amines still dominated as the primary adsorbent, as shown in Figure 14 [24]. Studies on functionalized MOF-177 also show that strongly polar groups are generally superior to -CH_3_ substitution in enhancing CO_2_ affinity [96]. The incorporation of alkyl chains and bulky non-polar substituents into metal–organic frameworks (MOFs) has been increasingly recognized as an effective molecular design strategy for regulating framework hydrophobicity. A representative example is provided by NMOF-1 decorated with exposed octadecyl chains, in which the long alkyl moieties substantially reduce the surface free energy and impart pronounced superhydrophobicity [97]. Importantly, the role of alkyl substitution is not limited to surface hydrophobization. Increasing evidence suggests that alkyl side chains can also profoundly affect framework assembly. For example, systematic extension of alkyl substituents has been shown to progressively increase the water contact angle from 136.9° to 155.0°, while the introduction of an n-octyl group can even drive a transformation from a three-dimensional framework to a two-dimensional layered structure [98]. A similar rationale applies to the use of bulky non-polar groups, whose steric and hydrophobic effects can further diversify pore environments. MOFs constructed from 5-tert-butylisophthalic acid have been reported to exhibit coexisting hydrophilic and hydrophobic channels, where the tert-butyl group serves as a sterically demanding hydrophobic unit [99]. These studies collectively emphasize that alkyl chain engineering and bulky nonpolar functionalization are versatile approaches for tuning the hydrophobicity, pore chemistry, and CO_2_ adsorption behavior of MOFs under humid conditions.

### 3.4. Synergistic Effects

Synergistic effects between metal doping or metal-node chemistry and functional groups are increasingly recognized as an effective strategy for improving CO_2_ physisorption and separation in MOFs. This is because the adsorption field is often governed by the combined influence of metal polarity, local coordination environment, and functional-group distribution, rather than by either factor alone. A simple example is that the same amino group can behave differently on different framework nodes. In aminated UiO-66 and Cu_3_(BTC)_2_, the CO_2_ uptakes at 298 K and 1 bar were 3.32 and 3.86 mmol g^−1^, respectively, but the CO_2_/N_2_ selectivities under a 15/85 vol% mixture were 120 for NH_2_-UiO-66 and 53 for NH_2_-Cu_3_(BTC)_2_, while NH_2_-Cu_3_(BTC)_2_ showed the highest Q_st_ of 43 kJ mol^−1^ [85]. These results show that the effect of a functional group depends strongly on the metal node and the surrounding pore environment.

More direct synergy has been reported when functional groups are introduced through reactive hydroxyl sites on metal clusters. In MOF-808, ethyleneamines were anchored through the μ-OH environment of the Zr cluster, and MOF-808-TEPA showed an IAST CO_2_/N_2_ selectivity of 256, about seven times that of pristine MOF-808, together with a CO_2_ uptake about 2.5 times higher at 15 kPa. A similar effect was observed in UiO-67, where aminosilanes introduced through μ-OH sites on the Zr node gave an IAST CO_2_/N_2_ selectivity of 407 at 100 kPa, 163 times that of pristine UiO-67, while the CO_2_ uptake at 15 kPa increased by about 2.6 times [100]. In these systems, the metal cluster not only supports the framework but also determines the accessibility and effective basicity of the amino groups.

Synergy is also seen when oxygen-containing groups act as secondary anchors or electronic modifiers near metal clusters. In the MOF-808 series, EDTA alone increased the CO_2_/N_2_ selectivity from 40 to 48, whereas reduced MOF-808-EDTA-ED-R reached 197, indicating stronger cooperation between oxygen-rich anchoring groups and newly formed amine sites. Nam et al. further showed that MOF808-EDTA-TREN reached a CO_2_/N_2_ selectivity of 519 at 100 kPa, although excessive functionalization greatly reduced the BET surface area and pore volume. A combined strategy was also reported for Mg-doped MOF-199, where Mg substitution increased CO_2_ uptake and subsequent polyethyleneimine loading further improved selectivity. Even without external amines, embedded functional groups can cooperate with metal centers, as shown in MFM-300(VIII), where bridging hydroxyl groups and V centers together strengthened CO_2_ binding. Overall, the strongest synergistic effects are currently found in systems combining moderate metal modification with amino or oxygen-containing groups, while comparable data for halogen–metal co-modification remain limited.

## 4. Computational Screening

Computational screening has evolved from a ranking exercise based mainly on single-component uptake and selectivity into a more demanding workflow that increasingly incorporates stability, humidity, and process-level relevance. Earlier high-throughput screening of experimental and hypothetical MOFs established the methodological basis, grand canonical Monte Carlo calculations enabled large-scale ranking, and early screening studies clarified that uptake alone is a poor decision variable. This remains an important point for modification studies. A modification that increases Q_st_ or low-pressure uptake may not improve working capacity or productivity in a pressure-swing process. Computational screening is therefore most useful when it translates local modifications into process-relevant descriptors rather than into a single scalar performance value [14,15,19,37,101,102,103,104,105,106,107,108].

Recent studies show a clear methodological refinement. Zhang et al. used machine learning to identify geometric targets for humid-condition CO_2_ capture and reported an optimal characteristic pore diameter around 14.18 Å and an accessible surface area around 1750 m^2^ g^−1^ [109], indicating that humid-flue-gas performance is associated with intermediate rather than extreme porosity. Kancharlapalli and Snurr extended this logic to wet flue gas through a multiscale screening strategy based on CoRE-MOF-2019 [18]. Mohamed et al. then moved the field further by integrating thermodynamic, mechanical, thermal, and activation stability metrics with high-throughput screening of hypothetical MOFs. In their study of 15,219 hMOFs, 148 candidates met the initial thresholds of CO_2_ uptake ≥4 mmol g^−1^ and CO_2_/N_2_ selectivity ≥200, and the highest CO_2_ uptake among those top-performing hMOFs was 8.47 mmol g^−1^ [19]. Screening studies now increasingly target realistic wet flue gas, identify hydrophobic or stability-compatible adsorption environments, and combine molecular simulation with data-driven models. Reviews by Demir et al. and Wang and Zhou emphasize that the next useful layer is process-aware design: screening should account for framework flexibility, defect chemistry, water competition, and the possibility that post-synthetic modification changes not only equilibrium loading but also diffusion and regenerability. Computational screening is most informative when it predicts which modification class is likely to help on a specific platform—for example, which pore diameter window benefits fluorination, which metal pair is likely to preserve topology in MOF-74, or what amine loading can be tolerated before pore blocking outweighs affinity gains. Hossein summarized recent research works on MD, GCMC, and DFT simulations of CO_2_ capture by MOFs [110].

## 5. Current Challenges and Future Perspectives

Despite rapid progress in tailoring MOFs for CO_2_ physisorption, the main challenge is no longer simply to maximize equilibrium uptake under ideal dry conditions, but to maintain working capacity, selectivity, and fast mass transfer under humid and compositionally complex gas streams. Many laboratory studies still evaluate adsorption mainly on activated powders under single-component or idealized binary conditions. In practice, however, successful separation requires reproducible synthesis, structured adsorbents, multicomponent breakthrough validation, effective heat management, and stable cyclic operation in the presence of water vapor. For physisorption-based CO_2_ separation, a moderate increase in host–guest affinity is valuable only if it does not greatly reduce pore accessibility, adsorption kinetics, or regeneration efficiency. Future experiments should therefore move beyond static uptake screening and focus more on standardized evaluation of working capacity, productivity, hydrothermal stability, shaping compatibility, and long-term cyclic durability. Recent progress in scalable and water-tolerant physisorbents is encouraging, but their practical value will depend on whether these properties can be preserved after pelletization, binder addition, and continuous operation under realistic flue-gas conditions [21].

From a theoretical perspective, materials are still often ranked by equilibrium uptake or ideal selectivity, whereas industrial separation is controlled by many coupled factors, including water competition, defect chemistry, framework flexibility, thermal effects, and process design. High-throughput screening and machine learning have pushed the field toward more realistic descriptors, but a clear gap remains between theory and experiment because simulations often assume ideal crystals, while real materials are defective, partially activated, and limited by shaping and cost [14]. The next stage should rely on a closed-loop workflow in which molecular simulation, process modeling, and experimental synthesis are integrated iteratively rather than treated separately. In this framework, the most useful models will not simply identify materials with the highest uptake, but predict frameworks that are synthetically accessible, mechanically robust, humidity-tolerant, and still competitive after shaping and under process-relevant conditions.

After industrial translation, economics will become the decisive factor. Process studies and production-cost analyses show that future success will depend less on record uptake values and more on whether a physisorbent can be made from low-cost precursors, processed into robust forms, and regenerated with low energy penalty while maintaining stable CO_2_/N_2_ separation performance [40]. Overall, recent advances in scalable physisorbents, robust Al-based frameworks, and process-integrated studies suggest that MOF-based CO_2_ physical separation is moving from proof-of-concept research toward practical deployment.

## 6. Conclusions

This review shows that aperture adjustment, metal-ion doping, and functional-group incorporation can each improve uptake or selectivity, but the gains are rarely independent. Stronger host–guest interactions often reduce accessible pore volume, slow diffusion, or raise regeneration energy. Accordingly, modified MOFs should not only be judged based on equilibrium capacity or equal heat dissipation, but also on working capacity, adsorption kinetics, cycle stability, water resistance, and energy consumption under real flue gas conditions. Aperture adjustment is best when moderate constraints and a favorable local electrostatic environment are introduced without significantly clogging the pores. Defect engineering can also be beneficial, but only within a narrow window in which additional binding sites are created without compromising crystallinity or framework robustness. Metal-ion doping shows particular promise in MOF-74 and related families, where controlled substitution can tune open-metal-site chemistry and channel polarity. Its benefit, however, depends on strict compositional control and retention of the parent structure. Functional-group incorporation, especially amine modification, remains the most reliable strategy for low-partial-pressure CO_2_ capture because it can enhance the interaction between CO_2_ and the skeleton. The main challenge is to avoid excessive loading, which can cause pore blocking, slower diffusion, and higher regeneration penalties, especially under humid and cyclic operation. Computational screening should be improved to enhance its practicality by incorporating framework flexibility, water competition, thermal stability, and synthetic feasibility. The most useful candidates for practical deployment will not necessarily be those with the highest reported uptake, but those that combine robust structure, reproducible synthesis, low regeneration cost, and stable performance over repeated cycles. This review proposes a clear MOF modification classification framework, providing guidance for the theoretical design of MOFs to improve CO_2_ adsorption capacity.

## Figures and Tables

**Table 1:** Adsorption performance of more MOFs with Aperture Adjustment.

Name	Index	Conditions	Value	Citation
PRI-1	CO_2_ adsorption	298 K, 1 bar	71.0 mg/g	[52]
Polymer-in-Cage ZIF-8	CO_2_/N_2_ Selectivity	298 K	80	[53]
Zn3 MOF	CO_2_/N_2_ Selectivity	298 K, 1.0 bar	4800	[54]
Cu-F-pymo	CO_2_/N_2_ Selectivity	298 K, 1.0 bar (15:85)	>10^7^	[55]
opt-UiO-66(Zr)-(OH)_2_	CO_2_ adsorption	298 K, 0.15 bar	2.50 mmol/g	[56]
UU-201	CO_2_ adsorption	293 K, 1.0 bar	3.52 mmol/g	[57]
MIL-120(Al)-AP	CO_2_/N_2_ Selectivity	298 K, 0.1 bar (15:85)	95	[41]
MOF-801(Ce)	CO_2_ adsorption	298 K, 1.0 bar	3.30 mmol/g	[58]
Zn-TCPP-dmtrz	CO_2_ adsorption	298 K, 1.0 bar	2.61 mmol/g	[59]
bio-MOF-12	CO_2_/N_2_ Selectivity	298 K, 1.0 bar (10:90)	52	[60]
Qc-5-Cu	CO_2_ adsorption	298 K, 1.0 bar	2.48 mmol/g	[61]
UiO-66@PAN10	CO_2_/N_2_ Selectivity	298 K (15:85)	17	[62]

**Table 2:** Summary of the improvement of CO_2_ adsorption performance by aperture adjustment.

Strategy	Structure Before and After Doping	Index	Values Before and After Doping	Citation
Linker skeleton engineering	MFM-136 → MFM-126	CO_2_/N_2_ selectivity	37.0 → 65.4	[63]
	[Zn_2_(bdc)_2_(dabco)] → [Zn_2_(tdc)_2_(dabco)]	CO_2_ uptake	46.0 → 67.4 cm^3^ g^−1^	[64]
	[Zn_2_(bdc)_2_(dabco)] → [Zn_2_(sedc)_2_(dabco)]	CO_2_/CH_4_ selectivity	11.9 → 15.1	[65]
Window-edge functionalization	DMOF-0F →DMOF-2F	CO_2_/N_2_ selectivity	12.4 → 21.9	[45]
	bio-MOF-11 → bio-MOF-12	CO_2_/N_2_ selectivity	73 → 123	[60]
Flexibility/gate-opening control	MOF-76-Ce →MOF-76-Ce	CO_2_ uptake	2.87 → 17.83 cm^3^ g^−1^	[66]
	closed-pore Fe(4-PyC)_2_(OH) → gate-open Fe(4-PyC)_2_(OH)	CO_2_/N_2_ selectivity	325 → 3131	[67]
	[Co_3_(OH)_2_(btca)_2_] → [Co_3_(OH)_2_(btca)_2_]·0.5DMF	CO_2_/N_2_ selectivity	46.3 → 79.6	[68]

**Table 3:** Representative examples of multimetallic doping in MOFs for CO_2_ adsorption and separation.

Strategy	Structure Before and After Doping	Values Before and After Doping	Index	Citation
Composition-balanced bimetallic synergy	Ni-MOF-74 → Ni_1_Co_1_-MOF-74	3.99 → 8.30 mmol g^−1^	CO_2_ uptake	[34]
	MIL-100(Fe) → MIL-100(Fe, Al)	2.60 → 3.27 mmol g^−1^	CO_2_ uptake	[75]
	ZIF-8-1000 → Zn/Ni-ZIF-8-1000	102 → 124	CO_2_/N_2_ selectivity	[76]
	CPM-200-In/Mg → CPM-200-Fe/Mg	190.9 → 207.6 cm^3^ g^−1^	CO_2_ uptake	[77]
	Ce-BTC → CuCe-BTC-1:2	0.10 → 0.74 mmol g^−1^	CO_2_ uptake	[78]
Heterometal substitution	MIL-96(Al) → MIL-96(Al)-Ca_1_	8.09 → 10.22 mmol g^−1^	CO_2_ uptake	[79]
	MIL-101(Cr) → MIL-101(Cr-Al)	1.82 → 4.2	CO_2_/CH_4_ selectivity	[67]
	Zn-MOF → Zn Ce-MOF	0.66 → 0.74 mmol g^−1^	CO_2_ uptake	[80]
	HKUST-1(Cu) → HKUST-1(Cu, Mg)	12.02 → 16.66	CO_2_/CH_4_ selectivity	[81]
	UiO-66(Zr) → Ti-exchanged UiO-66	2.3 → 4.0 mmol g^−1^	CO_2_ uptake	[82]
	MOF-5 → Cu0.05-MOF-5	3.52 → 4.61 mmol g^−1^	CO_2_ uptake	[83]

**Table 4:** Performances of some selected M808s in CO_2_ adsorptions at 298 K. (Reproduced with permission from reference [93].)

Material	CO_2_ Uptake (mmol/g)		Relative Adsorption Ratio *	CO_2_/N_2_ Selectivity **	CO_2_/N_2_ Selectivity **	−ΔH_st_ (kJ/mol) ***
	at 0.15 atm	at 1.0 atm		at 0.15 atm	at 1.0 atm	
M808	0.29	1.38	0.21	72	40	34
M808-EDTA	0.33	1.46	0.23	80	48	40
M808-EDTA-ED(0.6)	0.22	1.23	0.18	–	–	–
M808-EDTA-ED(1.2)	0.13	0.63	0.2	23	19	24
M808-EDTA-ED(1.8)	0.11	0.29	0.36	–	–	–
M808-EDTA-ED-R(1.2)	0.62	1.62	0.38	431	197	48

* Relative ratio of adsorption of 0.15 atm/1.0 atm. ** Calculated by IAST (for CO_2_:N_2_ = 0.15:0.75, at 1.0 atm and 298 K). *** Isosteric heat of adsorption at zero-coverage (calculated from the isotherms at 273, 288, 298 and 303 K).

**Table 5:** BET surface area (*S*_BET_), total pore volume (*V*_total_), isosteric heat of adsorption (*Q*_st_), and CO_2_/N_2_ selectivity (reproduced with permission from reference [45]).

	*S*_BET_ ^a^ (m^2^ g^−1^)	*V*_total_ ^b^ (cm^3^ g^−1^)	*Q*_st_ (kJ mol^−1^)	Selectivity (Initial Slope)	Selectivity ^c^ (IAST)
DMOF-0F	949	0.41	19.3	8.4	12.4
DMOF-1F	1123	0.48	20.2	11.3	14.5
DMOF-2F	1225	0.48	23.3	14.8	21.9

^a^ BET surface area. ^b^ Total pore volume. ^c^ Values at 0.3 bar.

## Data Availability

No new data were created or analyzed in this study.

## References

[1] Wang F., Harindintwali J.D., Wei K., Shan Y., Mi Z., Costello M.J., Grunwald S., Feng Z., Wang F., Guo Y. (2023). Climate Change: Strategies for Mitigation and Adaptation. Innov. Geosci..

[2] Lee H., Calvin K., Dasgupta D., Krinner G., Mukherji A., Thorne P.W., Trisos C., Romero J., Aldunce P., Barrett K. (2023). Climate Change 2023.

[3] Friedlingstein P., O’Sullivan M., Jones M.W., Andrew R.M., Hauck J., Landschützer P., Le Quéré C., Li H., Luijkx I.T., Olsen A. (2025). Global Carbon Budget 2024. Earth Syst. Sci. Data.

[4] Shu D.Y., Deutz S., Winter B.A., Baumgärtner N., Leenders L., Bardow A. (2023). The Role of Carbon Capture and Storage to Achieve Net-Zero Energy Systems: Trade-Offs between Economics and the Environment. Renew. Sustain. Energy Rev..

[5] Yamada H. (2020). Amine-Based Capture of CO_2_ for Utilization and Storage. Polym. J..

[6] Hou R., Fong C., Freeman B.D., Hill M.R., Xie Z. (2022). Current Status and Advances in Membrane Technology for Carbon Capture. Sep. Purif. Technol..

[7] Song C., Liu Q., Deng S., Li H., Kitamura Y. (2019). Cryogenic-Based CO_2_ Capture Technologies: State-of-the-Art Developments and Current Challenges. Renew. Sustain. Energy Rev..

[8] Blamey J., Anthony E.J., Wang J., Fennell P.S. (2010). The Calcium Looping Cycle for Large-Scale CO_2_ Capture. Prog. Energy Combust. Sci..

[9] Raganati F., Miccio F., Ammendola P. (2021). Adsorption of Carbon Dioxide for Post-Combustion Capture: A Review. Energy Fuels.

[10] Tao Z., Tian Y., Wu W., Liu Z., Fu W., Kung C.-W., Shang J. (2024). Development of Zeolite Adsorbents for CO_2_ Separation in Achieving Carbon Neutrality. npj Mater. Sustain..

[11] Ding M., Flaig R.W., Jiang H.L., Yaghi O.M. (2019). Carbon Capture and Conversion Using Metal-Organic Frameworks and Mof-Based Materials. Chem. Soc. Rev..

[12] Sun M., Wang X., Gao F., Xu M., Fan W., Xu B., Sun D. (2023). Synthesis Strategies of Metal-Organic Frameworks for CO_2_ Capture. Microstructures.

[13] Ding M., Rong W., Wang Y., Kong S., Yao J. (2023). Pore Engineering of Metal–Organic Frameworks for Boosting Low-Pressure CO_2_ Capture. J. Mater. Chem. A.

[14] Demir H., Daglar H., Gulbalkan H.C., Aksu G.O., Keskin S. (2023). Recent Advances in Computational Modeling of Mofs: From Molecular Simulations to Machine Learning. Coord. Chem. Rev..

[15] Wang Z., Zhou T. (2025). Computer-Aided Metal–Organic Framework Screening and Design Approaches toward Efficient Carbon Capture Processes. Mol. Syst. Des. Eng..

[16] Choe J.H., Kim H., Hong C.S. (2021). Mof-74 Type Variants for CO_2_ Capture. Mater. Chem. Front..

[17] Chung Y.G., Haldoupis E., Bucior B.J., Haranczyk M., Lee S., Zhang H., Vogiatzis K.D., Milisavljevic M., Ling S., Camp J.S. (2019). Advances, Updates, and Analytics for the Computation-Ready, Experimental Metal–Organic Framework Database: Core Mof 2019. J. Chem. Eng. Data.

[18] Kancharlapalli S., Snurr R.Q. (2023). High-Throughput Screening of the Core-Mof-2019 Database for CO_2_ Capture from Wet Flue Gas: A Multi-Scale Modeling Strategy. ACS Appl. Mater. Interfaces.

[19] Mohamed S.A., Zhao D., Jiang J. (2023). Integrating Stability Metrics with High-Throughput Computational Screening of Metal–Organic Frameworks for CO_2_ Capture. Commun. Mater..

[20] Mahajan S., Lahtinen M. (2022). Recent Progress in Metal-Organic Frameworks (Mofs) for CO_2_ Capture at Different Pressures. J. Environ. Chem. Eng..

[21] Siegelman R.L., Kim E.J., Long J.R. (2021). Porous Materials for Carbon Dioxide Separations. Nat. Mater..

[22] Li J.R., Sculley J., Zhou H.C. (2012). Metal-Organic Frameworks for Separations. Chem. Rev..

[23] Zhou L., Niu Z., Jin X., Tang L., Zhu L. (2018). Effect of Lithium Doping on the Structures and CO_2_ Adsorption Properties of Metal-Organic Frameworks Hkust-1. ChemistrySelect.

[24] Lee G., Jhung S.H. (2025). CO_2_ Capture Using Functionalized Mil-101(Cr) Metal–Organic Frameworks: Functionality Nanoarchitectonics of Nanospace for CO_2_ Adsorption. Sep. Purif. Technol..

[25] Song L., Xue C., Xia H., Qiu S., Sun L., Chen H. (2019). Effects of Alkali Metal (Li, Na, and K) Incorporation in NH_2_-Mil_125_Ti on the Performance of CO_2_ Adsorption. Materials.

[26] Huang Z., Ying L., Gong F., Lu J., Wang W., Ding J., Yan J. (2023). Aminosilane-Functionalized Ti-Based Metal–Organic Framework for Efficient and Selective CO_2_ Adsorption. J. Environ. Chem. Eng..

[27] Ma M., Zhou A., Hong T., Jia X., Liu M. (2023). Tailored Porous Structure and CO_2_ Adsorption Capacity of Mg-Mof-74 Via Solvent Polarity Regulation. Chem. Eng. J..

[28] Xin C., Hou S., Yu L., Zhou X., Fu Y., Yang X., Sun W., Yang F., Wang X., Liu L. (2024). Controlled Synthesis of Mg-Mof-74 and Its CO_2_ Adsorption in Flue Gas. Coatings.

[29] An H., Tian W., Lu X., Yuan H., Yang L., Zhang H., Shen H., Bai H. (2023). Boosting the CO_2_ Adsorption Performance by Defect-Rich Hierarchical Porous Mg-Mof-74. Chem. Eng. J..

[30] Kazemi A., Pordsari M.A., Tamtaji M., Zainali F., Keshavarz S., Baesmat H., Manteghi F., Ghaemi A., Rohani S., Goddard W.A. (2025). Eco-Friendly Synthesis and Morphology Control of Mof-74 for Exceptional CO_2_ Capture Performance with Dft Validation. Sep. Purif. Technol..

[31] Shi X.-R., Qiao M., Wei Y., Yun L.-X., Wang J.-X., Chen J.-F. (2024). Green, Efficient and Controllable Synthesis of High-Quality Mof-74 with High Gravity Technology. Green Chem..

[32] Cui S., Gu Y., Shao Y., Zhong W. (2024). Experimental Research of Alkali Metals Modified Mg/Dobdc Metal Organic Framework as High Capacity CO_2_ Adsorbent. Sep. Purif. Technol..

[33] Ullah S., Bustam M.A., Sagir M., Raza M.R., Nawaz S., Assiri M.A., Al-Sehemi A.G., Mukhtar A., Feng C.L., Ayoub M. (2025). Synthesis and Doping of Alkali Metals on Mof-74 for CO_2_ and CH_4_ Pure and Binary Mixtures Adsorption. Gas Sci. Eng..

[34] Chen C., Kosari M., Jing M., He C. (2022). Microwave-Assisted Synthesis of Bimetallic Nico-Mof-74 with Enhanced Open Metal Site for Efficient CO_2_ Capture. Environ. Funct. Mater..

[35] Kazemi A., Pordsari M.A., Tamtaji M., Manteghi F., Ghaemi A., Rohani S., Goddard W.A. (2025). Environmentally Friendly Synthesis and Morphology Engineering of Mixed-Metal Mof for Outstanding CO_2_ Capture Efficiency. Chem. Eng. J..

[36] Zhang X., Li G., Hong M., Ban H., Yang L., Liu X., Li F., Matus E.V., Li C., Li L. (2024). Development of Ni/Mg_1_-Mof-74 for Highly Efficient CO_2_/N_2_ Separation. J. Fuel Chem. Technol..

[37] Farmahini A.H., Krishnamurthy S., Friedrich D., Brandani S., Sarkisov L. (2021). Performance-Based Screening of Porous Materials for Carbon Capture. Chem. Rev..

[38] Ahlen M., Cheung O., Xu C. (2023). Low-Concentration CO_2_ Capture Using Metal-Organic Frameworks-Current Status and Future Perspectives. Dalton Trans..

[39] Kazemi A., Moghadaskhou F., Pordsari M.A., Manteghi F., Tadjarodi A., Ghaemi A. (2023). Enhanced CO_2_ Capture Potential of Uio-66-NH_2_ Synthesized by Sonochemical Method: Experimental Findings and Performance Evaluation. Sci. Rep..

[40] Peh S.B., Farooq S., Zhao D. (2023). Techno-Economic Analysis of Mof-Based Adsorption Cycles for Postcombustion CO_2_ Capture from Wet Flue Gas. Chem. Eng. Sci..

[41] Chen B., Fan D., Pinto R.V., Dovgaliuk I., Nandi S., Chakraborty D., Garcia-Moncada N., Vimont A., McMonagle C.J., Bordonhos M. (2024). A Scalable Robust Microporous Al-Mof for Post-Combustion Carbon Capture. Adv. Sci..

[42] Siegelman R.L., Milner P.J., Forse A.C., Lee J.H., Colwell K.A., Neaton J.B., Reimer J.A., Weston S.C., Long J.R. (2019). Water Enables Efficient CO_2_ Capture from Natural Gas Flue Emissions in an Oxidation-Resistant Diamine-Appended Metal-Organic Framework. J. Am. Chem. Soc..

[43] Alivand M.S., Mazaheri O., Wu Y., Zavabeti A., Christofferson A.J., Meftahi N., Russo S.P., Stevens G.W., Scholes C.A., Mumford K.A. (2022). Engineered Assembly of Water-Dispersible Nanocatalysts Enables Low-Cost and Green CO_2_ Capture. Nat. Commun..

[44] Lee G., Ahmed I., Jhung S.H. (2024). CO_2_ Adsorption Using Functionalized Metal–Organic Frameworks under Low Pressure: Contribution of Functional Groups, Excluding Amines, to Adsorption. Chem. Eng. J..

[45] Di T., Yoshida Y., Otake K.I., Kitagawa S., Kitagawa H. (2024). Increased CO_2_/N_2_ Selectivity by Stepwise Fluorination in Isoreticular Ultramicroporous Metal-Organic Frameworks. Chem. Sci..

[46] Venturi D.M., Notari M.S., Bondi R., Mosconi E., Kaiser W., Mercuri G., Giambastiani G., Rossin A., Taddei M., Costantino F. (2022). Increased CO_2_ Affinity and Adsorption Selectivity in Mof-801 Fluorinated Analogues. ACS Appl. Mater. Interfaces.

[47] Kazemi A., Pordsari M.A., Tamtaji M., Afshari M.H., Keshavarz S., Zeinali F., Baesmat H., Zahiri S., Manteghi F., Ghaemi A. (2024). Unveiling the Power of Defect Engineering in Mof-808 to Enhance Efficient Carbon Dioxide Adsorption and Separation by Harnessing the Potential of Dft Analysis. Chem. Eng. J..

[48] Agueda V.I., Delgado J.A., Uguina M.A., Brea P., Spjelkavik A.I., Blom R., Grande C. (2015). Adsorption and Diffusion of H_2_, N_2_, CO, CH_4_ and CO_2_ in Utsa-16 Metal-Organic Framework Extrudates. Chem. Eng. Sci..

[49] Gaikwad S., Han S. (2023). Shaping Metal-Organic Framework (Mof) with Activated Carbon and Silica Powder Materials for CO_2_ Capture. J. Environ. Chem. Eng..

[50] Grande C.A., Blom R., Middelkoop V., Matras D., Vamvakeros A., Jacques S.D.M., Beale A.M., Di Michiel M., Anne Andreassen K., Bouzga A.M. (2020). Multiscale Investigation of Adsorption Properties of Novel 3d Printed Utsa-16 Structures. Chem. Eng. J..

[51] Henrotin A., Heymans N., Duprez M.E., Mouchaham G., Serre C., Wong D., Robinson R., Mulrooney D., Casaban J., De Weireld G. (2024). Lab-Scale Pilot for CO_2_ Capture Vacuum Pressure Swing Adsorption: Mil-160(Al) Vs Zeolite 13x. Carbon Capture Sci. Technol..

[52] Li Y., Bai Y., Wang Z., Gong Q., Li M., Bo Y., Xu H., Jiang G., Chi K. (2023). Exquisitely Constructing a Robust Mof with Dual Pore Sizes for Efficient CO_2_ Capture. Molecules.

[53] Kim M., Min H.J., Choi M.K., Kim K.C., Kim B., Kang M., Lee N., Eum K., Kim J.H., Kim D.W. (2026). Polymer-in-Cage Strategy for Pore Tuning of High-Aspect Ratio Zif Nanoplate: Toward Sub-Micrometer-Thick Large Area CO_2_ Separation Membranes. Adv. Sci..

[54] Berdichevsky E.K., Downing V.A., Hooper R.W., Butt N.W., McGrath D.T., Donnelly L.J., Michaelis V.K., Katz M.J. (2022). Ultrahigh Size Exclusion Selectivity for Carbon Dioxide from Nitrogen/Methane in an Ultramicroporous Metal-Organic Framework. Inorg. Chem..

[55] Shi Y., Xie Y., Cui H., Alothman Z.A., Alduhaish O., Lin R.-B., Chen B. (2022). An Ultramicroporous Metal–Organic Framework with Dual Functionalities for High Sieving Separation of CO_2_ from CH_4_ and N_2_. Chem. Eng. J..

[56] Hu Z., Wang Y., Farooq S., Zhao D. (2017). A Highly Stable Metal-Organic Framework with Optimum Aperture Size for CO_2_ Capture. AIChE J..

[57] Åhlén M., Zhou Y., Hedbom D., Cho H.S., Strømme M., Terasaki O., Cheung O. (2023). Efficient SF_6_ capture and separation in robust gallium- and vanadium-based metal–organic frameworks. J. Mater. Chem. A.

[58] Li C.N., Wang S.M., Tao Z.P., Liu L., Xu W.G., Gu X.J., Han Z.B. (2023). Green Synthesis of Mof-801(Zr/Ce/Hf) for CO_2_/N_2_ and CO_2_/CH_4_ Separation. Inorg. Chem..

[59] Zhu Z., Xiao J., Zhang M., Huang Y., Yuan S. (2025). Precision Pore Engineering Via Fit-Topology Assembly in a Zn-Porphyrin Mof for Selective C_2_H_2_ Capture. Chem. Sci..

[60] Li T., Chen D.-L., Sullivan J.E., Kozlowski M.T., Johnson J.K., Rosi N.L. (2013). Systematic Modulation and Enhancement of CO_2_: N_2_ Selectivity and Water Stability in an Isoreticular Series of Bio-Mof-11 Analogues. Chem. Sci..

[61] He T., Xiao Y., Zhao Q., Zhou M., He G. (2020). Ultramicroporous Metal–Organic Framework Qc-5-Cu for Highly Selective Adsorption of CO_2_ from C_2_H_4_ Stream. Ind. Eng. Chem. Res..

[62] Gebremariam S.K., Varghese A.M., Ehrling S., Al Wahedi Y., AlHajaj A., Dumee L.F., Karanikolos G.N. (2024). Hierarchically Porous Structured Adsorbents with Ultrahigh Metal-Organic Framework Loading for CO_2_ Capture. ACS Appl. Mater. Interfaces.

[63] Humby J.D., Benson O., Smith G.L., Argent S.P., da Silva I., Cheng Y., Rudic S., Manuel P., Frogley M.D., Cinque G. (2019). Host-Guest Selectivity in a Series of Isoreticular Metal-Organic Frameworks: Observation of Acetylene-to-Alkyne and Carbon Dioxide-to-Amide Interactions. Chem. Sci..

[64] Bolotov V.A., Kovalenko K.A., Samsonenko D.G., Han X., Zhang X., Smith G.L., McCormick L.J., Teat S.J., Yang S., Lennox M.J. (2018). Enhancement of CO_2_ Uptake and Selectivity in a Metal-Organic Framework by the Incorporation of Thiophene Functionality. Inorg. Chem..

[65] Demakov P.A., Volynkin S.S., Samsonenko D.G., Fedin V.P., Dybtsev D.N. (2020). A Selenophene-Incorporated Metal-Organic Framework for Enhanced CO_2_ Uptake and Adsorption Selectivity. Molecules.

[66] Ethiraj J., Bonino F., Vitillo J.G., Lomachenko K.A., Lamberti C., Reinsch H., Lillerud K.P., Bordiga S. (2016). Solvent-Driven Gate Opening in Mof-76-Ce: Effect on CO_2_ Adsorption. ChemSusChem.

[67] Singh H.D., Nandi S., Chakraborty D., Singh K., Vinod C.P., Vaidhyanathan R. (2022). Coordination Flexibility Aided CO_2_ -Specific Gating in an Iron Isonicotinate Mof. Chem. Asian J..

[68] Ren H.-Y., Zhang X.-M. (2015). Enhanced Selective CO_2_ Capture Upon Incorporation of Dimethylformamide in the Cobalt Metal–Organic Framework [CO_3_(OH)_2_(Btca)_2_]. Energy Fuels.

[69] Ullah Khan A., Samuel O., Othman M.H.D., Younas M., Kamaludin R., Puteh M.H., Kurniawan T.A., Yinn Wong K., Kadirkhan F., Yoshida N. (2024). Enhancing CO_2_ Adsorption Selectivity of Mof-199: Investigating the Synergistic Effect of Mg Metal Doping and Polyethyleneimine Impregnation. Sep. Purif. Technol..

[70] Liu J., Tian J., Thallapally P.K., McGrail B.P. (2012). Selective CO_2_ Capture from Flue Gas Using Metal–Organic Frameworks―A Fixed Bed Study. J. Phys. Chem. C.

[71] Remy T., Peter S.A., Van der Perre S., Valvekens P., De Vos D.E., Baron G.V., Denayer J.F.M. (2013). Selective Dynamic CO_2_ Separations on Mg-Mof-74 at Low Pressures: A Detailed Comparison with 13×. J. Phys. Chem. C.

[72] Liu J., Wang Y., Benin A.I., Jakubczak P., Willis R.R., LeVan M.D. (2010). CO_2_/H_2_O Adsorption Equilibrium and Rates on Metal-Organic Frameworks: Hkust-1 and Ni/Dobdc. Langmuir.

[73] Cui S., Shao Y., Zhong W. (2023). Synthesis and Characterization of Novel Bimetallic Mg-Ca/Dobdc Metal—Organic Frameworks as a High Stability CO_2_ Adsorbent. Chem. Eng. J..

[74] Rafati Jolodar A., Abdollahi M., Fatemi S., Mansoubi H. (2024). Enhancing Carbon Dioxide Separation from Natural Gas in Dynamic Adsorption by a New Type of Bimetallic Mof; MIL-101(Cr-Al). Sep. Purif. Technol..

[75] Le V.N., Nguyen V.C., Nguyen H.T., Tran H.D., Tu T.N., Kim W.-S., Kim J. (2023). Facile Synthesis of Bimetallic Mil-100(Fe, Al) for Enhancing CO_2_ Adsorption Performance. Microporous Mesoporous Mater..

[76] Li R., Ren X., Feng X., Li X., Hu C., Wang B. (2014). A Highly Stable Metal- and Nitrogen-Doped Nanocomposite Derived from Zn/Ni-ZIF-8 Capable of CO_2_ Capture and Separation. Chem. Commun..

[77] Raptopoulou C.P. (2021). Metal-Organic Frameworks: Synthetic Methods and Potential Applications. Materials.

[78] Jampaiah D., Shah D., Chalkidis A., Saini P., Babarao R., Arandiyan H., Bhargava S.K. (2024). Bimetallic Copper-Cerium-Based Metal-Organic Frameworks for Selective Carbon Dioxide Capture. Langmuir.

[79] Abid H.R., Rada Z.H., Li Y., Mohammed H.A., Wang Y., Wang S., Arandiyan H., Tan X., Liu S. (2020). Boosting CO_2_ Adsorption and Selectivity in Metal-Organic Frameworks of MIL-96(Al) Via Second Metal Ca Coordination. RSC Adv..

[80] Cui P., Tang Y., Guo A., Wang C., Liu M., Peng W., Yu F. (2024). Enhanced CO_2_ Adsorption Properties with Bimetallic Znce-Mof Prepared Using a Microchannel Reactor. Front. Chem. Sci. Eng..

[81] Denning S., Majid A.A., Lucero J.M., Crawford J.M., Carreon M.A., Koh C.A. (2020). Metal-Organic Framework Hkust-1 Promotes Methane Hydrate Formation for Improved Gas Storage Capacity. ACS Appl. Mater. Interfaces.

[82] Lau C.H., Babarao R., Hill M.R. (2013). A Route to Drastic Increase of CO_2_ Uptake in Zr Metal Organic Framework Uio-66. Chem. Commun..

[83] Lei R., Shen W., Yang Z., Jin H., Chai W., Guo X., Ge H., Jin D. (2025). Enhanced CO_2_ Adsorption in Cux-Mof-5: Optimal Doping and Regeneration Performance. Mater. Today Sustain..

[84] Mutyala S., Yu Y.-D., Jin W.-G., Wang Z.-S., Zheng D.-Y., Ye C.-R., Luo B. (2019). CO_2_ Capture Using Amine Incorporated Uio-66 in Atmospheric Pressure. J. Porous Mater..

[85] Pirzadeh K., Esfandiari K., Ghoreyshi A.A., Rahimnejad M. (2020). CO_2_ and N_2_ Adsorption and Separation Using Aminated Uio-66 and Cu_3_(Btc)_2_: A Comparative Study. Korean J. Chem. Eng..

[86] Gaikwad S., Kim Y., Gaikwad R., Han S. (2021). Enhanced CO_2_ Capture Capacity of Amine-Functionalized Mof-177 Metal Organic Framework. J. Environ. Chem. Eng..

[87] Jun H.J., Yoo D.K., Jhung S.H. (2022). Metal-Organic Framework (Mof-808) Functionalized with Ethyleneamines: Selective Adsorbent to Capture CO_2_ under Low Pressure. J. CO2 Util..

[88] Nam H.Y., Lee G., Jhung S.H. (2024). Selective CO_2_ Adsorption over a Zr-Based Metal–Organic Framework Functionalized with Tris(2-Aminoethyl)Amine. Chem. Eng. J..

[89] Esfahani H.J., Shahhosseini S., Ghaemi A. (2023). Improved Structure of Zr-Btc Metal Organic Framework Using NH2 to Enhance CO_2_ Adsorption Performance. Sci. Rep..

[90] Quan W., Holmes H.E., Zhang F., Hamlett B.L., Finn M.G., Abney C.W., Kapelewski M.T., Weston S.C., Lively R.P., Koros W.J. (2022). Scalable Formation of Diamine-Appended Metal-Organic Framework Hollow Fiber Sorbents for Postcombustion CO_2_ Capture. JACS Au.

[91] Hughes R., Yancy-Caballero D., Zamarripa-Perez M., Omell B., Matuszewski M., Bhattacharyya D. (2024). Modeling and Techno-Economic Optimization of a Tetraamine-Appended Metal–Organic Framework for Ngcc-Based CO_2_ Capture Using Fixed Bed Contactors. Energy Fuels.

[92] Lu Z., Godfrey H.G., da Silva I., Cheng Y., Savage M., Tuna F., McInnes E.J., Teat S.J., Gagnon K.J., Frogley M.D. (2017). Modulating Supramolecular Binding of Carbon Dioxide in a Redox-Active Porous Metal-Organic Framework. Nat. Commun..

[93] Park J.M., Yoo D.K., Jhung S.H. (2020). Selective CO_2_ Adsorption over Functionalized Zr-Based Metal Organic Framework under Atmospheric or Lower Pressure: Contribution of Functional Groups to Adsorption. Chem. Eng. J..

[94] Finsy V., Ma L., Alaerts L., De Vos D.E., Baron G.V., Denayer J.F.M. (2009). Separation of CO_2_/CH_4_ Mixtures with the MIL-53(Al) Metal–Organic Framework. Microporous Mesoporous Mater..

[95] Han Y., Ji C., Lou Y., Li R., He M., Yuan D., Han Z. (2025). Topology-Directed Cage Engineering in Mofs for Efficient C_2_H_2_/CO_2_/C_2_H_4_ Separation. J. Am. Chem. Soc..

[96] Gu C., Liu Y., Wang W., Liu J., Hu J. (2020). Effects of Functional Groups for CO_2_ Capture Using Metal Organic Frameworks. Front. Chem. Sci. Eng..

[97] Roy S., Suresh V.M., Maji T.K. (2016). Self-Cleaning Mof: Realization of Extreme Water Repellence in Coordination Driven Self-Assembled Nanostructures. Chem. Sci..

[98] He X., Yu C., Li J., Wang Z., Ye K.-Y. (2024). Introducing Alkyl Chains to Realize the Construction of Superhydrophobic/Superoleophilic Mofs and the Transformation from Three-Dimensional to Two-Dimensional Structure. Inorg. Chem. Front..

[99] Chaudhari A.K., Mukherjee S., Nagarkar S.S., Joarder B., Ghosh S.K. (2013). Bi-Porous Metal–Organic Framework with Hydrophilic and Hydrophobic Channels: Selective Gas Sorption and Reversible Iodine Uptake Studies. CrystEngComm.

[100] Yoo D.K., Jhung S.H. (2022). Selective CO_2_ Adsorption at Low Pressure with a Zr-Based Uio-67 Metal–Organic Framework Functionalized with Aminosilanes. J. Mater. Chem. A.

[101] Pham T., Space B. (2020). Insights into the Gas Adsorption Mechanisms in Metal-Organic Frameworks from Classical Molecular Simulations. Top. Curr. Chem..

[102] Colon Y.J., Snurr R.Q. (2014). High-Throughput Computational Screening of Metal-Organic Frameworks. Chem. Soc. Rev..

[103] Qiao Z., Zhang K., Jiang J. (2016). In Silico Screening of 4764 Computation-Ready, Experimental Metal–Organic Frameworks for CO_2_ Separation. J. Mater. Chem. A.

[104] Li S., Chung Y.G., Snurr R.Q. (2016). High-Throughput Screening of Metal-Organic Frameworks for CO_2_ Capture in the Presence of Water. Langmuir.

[105] Qiao Z., Peng C., Zhou J., Jiang J. (2016). High-Throughput Computational Screening of 137953 Metal–Organic Frameworks for Membrane Separation of a CO_2_/N_2_/CH_4_ Mixture. J. Mater. Chem. A.

[106] Ji C., Zhang K. (2024). Computational Screening of Metal–Organic Frameworks for Separation of CO_2_ and N_2_ from Wet Flue Gas. J. Mater. Sci..

[107] Boyd P.G., Chidambaram A., Garcia-Diez E., Ireland C.P., Daff T.D., Bounds R., Gladysiak A., Schouwink P., Moosavi S.M., Maroto-Valer M.M. (2019). Data-Driven Design of Metal-Organic Frameworks for Wet Flue Gas CO_2_ Capture. Nature.

[108] Daglar H., Keskin S. (2020). Recent Advances, Opportunities, and Challenges in High-Throughput Computational Screening of Mofs for Gas Separations. Coord. Chem. Rev..

[109] Zhang X., Zhang K., Yoo H., Lee Y. (2021). Machine Learning-Driven Discovery of Metal–Organic Frameworks for Efficient CO_2_ Capture in Humid Condition. ACS Sustain. Chem. Eng..

[110] Mashhadimoslem H., Abdol M.A., Karimi P., Zanganeh K., Shafeen A., Elkamel A., Kamkar M. (2024). Computational and Machine Learning Methods for CO_2_ Capture Using Metal-Organic Frameworks. ACS Nano.

